# Patient-Wise Methodology to Assess Glycemic Health Status: Applications to Quantify the Efficacy and Physiological Targets of Polyphenols on Glycemic Control

**DOI:** 10.3389/fnut.2022.831696

**Published:** 2022-02-17

**Authors:** Álvaro Olivera-Nappa, Sebastian Contreras, María Florencia Tevy, David Medina-Ortiz, Andrés Leschot, Pilar Vigil, Carlos Conca

**Affiliations:** ^1^Centre for Biotechnology and Bioengineering (CeBiB), University of Chile, Santiago, Chile; ^2^Department of Chemical Engineering, Biotechnology and Materials, University of Chile, Santiago, Chile; ^3^Max Planck Institute for Dynamics and Self-Organization, Göttingen, Germany; ^4^Laboratory of Cell Biology, Institute of Nutrition and Food Technology (INTA), University of Chile, Santiago, Chile; ^5^Maqui New Life S.A., Santiago, Chile; ^6^Reproductive Health Research Institute, Santiago, Chile; ^7^Center for Mathematical Modelling (CMM), University of Chile, Santiago, Chile

**Keywords:** dysglycemia risk, glycemia, precision preventive medicine, early disglycemia diagnosis, delphinol polyphenols, quantitative diagnostic, oral glucose tolerance test (OGTT)

## Abstract

A growing body of evidence indicates that dietary polyphenols could be used as an early intervention to treat glucose-insulin (G-I) dysregulation. However, studies report heterogeneous information, and the targets of the intervention remain largely elusive. In this work, we provide a general methodology to quantify the effects of any given polyphenol-rich food or formulae over glycemic regulation in a patient-wise manner using an Oral Glucose Tolerance Test (OGTT). We use a mathematical model to represent individual OGTT curves as the coordinated action of subsystems, each one described by a parameter with physiological interpretation. Using the parameter values calculated for a cohort of 1198 individuals, we propose a statistical model to calculate the risk of dysglycemia and the coordination among subsystems for each subject, thus providing a continuous and individual health assessment. This method allows identifying individuals at high risk of dysglycemia—which would have been missed with traditional binary diagnostic methods—enabling early nutritional intervention with a polyphenol-supplemented diet where it is most effective and desirable. Besides, the proposed methodology assesses the effectiveness of interventions over time when applied to the OGTT curves of a treated individual. We illustrate the use of this method in a case study to assess the dose-dependent effects of Delphinol® on reducing dysglycemia risk and improving the coordination between subsystems. Finally, this strategy enables, on the one hand, the use of low-cost, non-invasive methods in population-scale nutritional studies. On the other hand, it will help practitioners assess the effectiveness of an intervention based on individual vulnerabilities and adapt the treatment to manage dysglycemia and avoid its progression into disease.

## 1. Introduction

Disequilibrium in glucose-insulin (G-I) homeostasis (dysglycemia) is a widespread condition in modern society and is associated with poor dietary habits and poverty (1–4). Dysglycemia may progress through insulin resistance and glucose intolerance to type 2 diabetes mellitus (T2DM), and increase the risk for cardiovascular diseases and other comorbidities. Non-pharmacological preventive interventions such as diet supplemented with (or rich in) polyphenols may be crucial to avoid the progression of a dysglycemic condition into a disease state (5–10).

Polyphenols constitute a category of more than 500 compounds divided into six subclasses (http://phenol-explorer.eu/). In particular, anthocyanin-rich foods and extracts are becoming a mainstay in fighting early dysglycemia and its related health conditions (11–13). Current research suggests that polyphenolic molecules' effects on glucose metabolism are pleiotropic, multi-targeted, and synergistic (9, 10, 14–21). However, despite the abundance of knowledge about molecular targets and mechanisms of action affected by the intake of polyphenols, there is no consensus on which types of polyphenolic compounds target which mechanisms and to what extent. Hence, there is a need for quantitative methods to address such questions and follow the effects of any given polyphenolic formula over glycemic regulation to advance towards standardisation and comparison, particularly in clinical cohort results. Furthermore, such methods must be sensitive enough to detect subtle changes to keep track of the nutritional treatment since dietary interventions are often subtle and long term (22).

**Figure 1:**
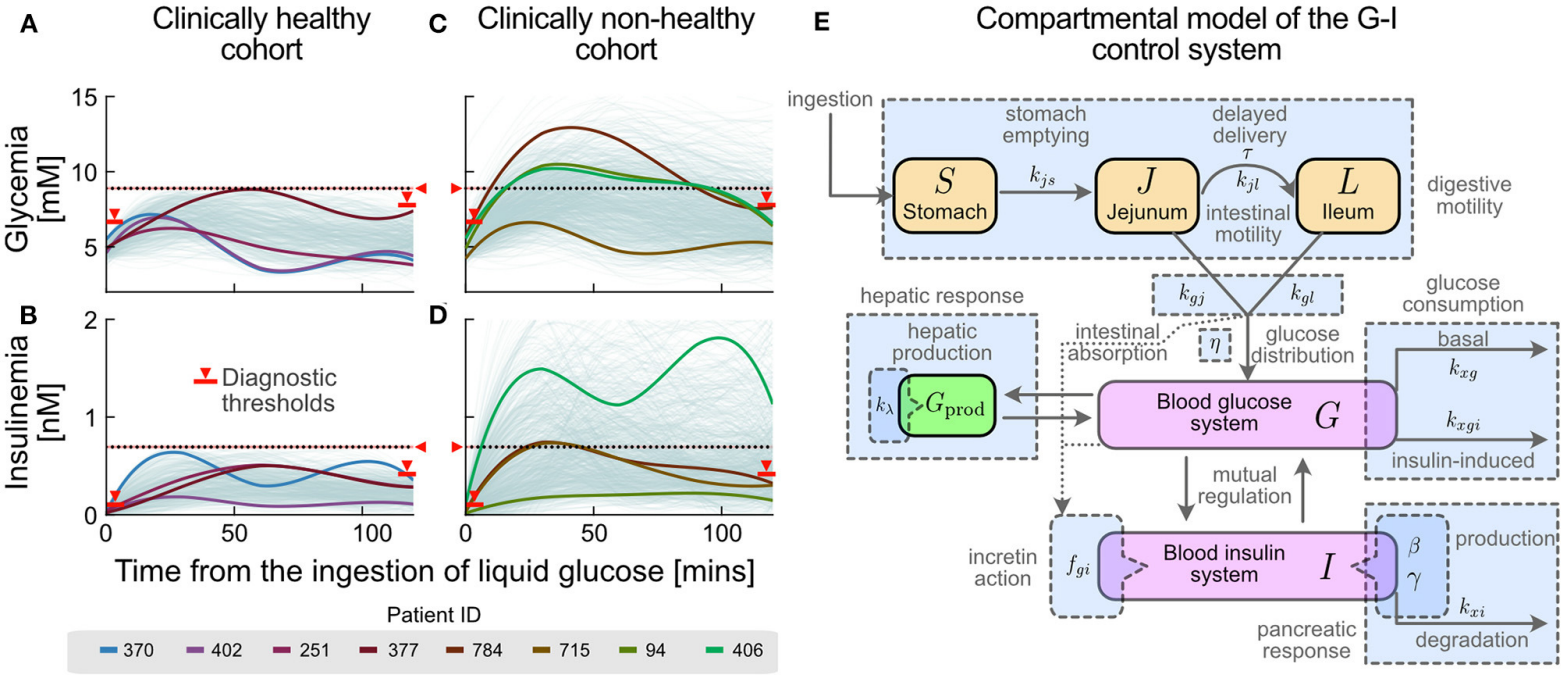
Different glucose-insulin (G-I) curve shapes are masked under the current clinical criteria of normality. Current clinical criteria classify an individual as healthy **(A,B)** or non-healthy **(C,D)** based on threshold values for their OGTT response (see Methods and Supplementary Table S1). Red markers and horizontal lines in both glycemia-insulinemia represent current criteria of normality for an OGTT, including basal (pre-prandial), final (2-hr) and all-time maxima. Different colours represent selected individual subjects in the healthy **(A,B)** and non-healthy **(C,D)** cohorts whose glycemia curves are similar, but having different insulinemia curves (subjects 370/402 and 94/406), or similar insulinemia curves but different glycemia curves (subject 251/377 and 784/715). **(E)** G-I dynamics during an OGTT can be represented by a compact compartmental mathematical model (23), where the main physiological processes in G-I dynamics are represented as subsystems. Compartments are represented by coloured boxes (orange for glucose pools in the lumen of the digestive system, magenta for blood concentrations and green for the liver compartment). Rate constants and parameters for each physiological process are indicated alongside their respective process arrows or compartments. General related processes, which account for subsystems, are shown as blue boxes.

Mathematical modelling has long been used to quantify the G-I function. We recently presented a model (23) representing G-I dynamics as a set of separate coordinated subsystems, each characterised by quantitative physiological parameters. Parameters are obtained in a patient-wise manner from a low-cost, non-invasive, routine Oral Glucose Tolerance Test (OGTT) with insulinemia measurements at 5-time points, instead of the standard 2 points (cf. Figures 1A–D for examples of these curves). The model considers five compartments for describing G-I dynamics through coordinated subsystems coupled *via* parameters with physiological meaning, as represented in Figure 1E. Such compartments represent the insulinemia (I), and the concentration or amount of glucose in different compartments: in the stomach S, in the upper intestinal tract J (jejunum) and L (ileum), and the bloodstream (glycemia) G. Relevant subsystems are highlighted with light blue. Thus, the effects of a polyphenolic intervention on G-I dynamics may be dissected and compared among individuals or cohorts using this model.

In this work, we propose a general methodology to assess glycemic health status and dysglycemia risk in a patient-wise manner and apply it to quantify the effectiveness of polyphenolic supplementation in the diet at a cohort or individual level. The novel aspects of the present work are related to (a) the application of the mathematical model presented in Contreras et al. (23) on a large cohort, (b) the statistical analysis of cohort parametric values obtained by fitting the model to each subject's glycemia and insulinemia OGTT curves, (c) the development of non-dimensional numbers (NDNs) able to express each patient's own OGTT dynamics in a comparable dynamic scale, independent of each subject's status and particularities, (d) the definition and calculation of normal, undesirable and abnormal ranges for each parameter, (e) the development of a statistical model to assign dysglycemia risk to individuals based on current definitions of glycemic health, (f) the description of the correlation between parameters/NDNs and the dis-coordination found in non-healthy states, (g) the application of the former findings to nutritional interventions involving a polyphenol-rich supplement (Delphinol®), and (h) the dose-dependent effects of Delphinol® on each of the OGTT parameters (all from a clinical point of view).

**Figure 2:**
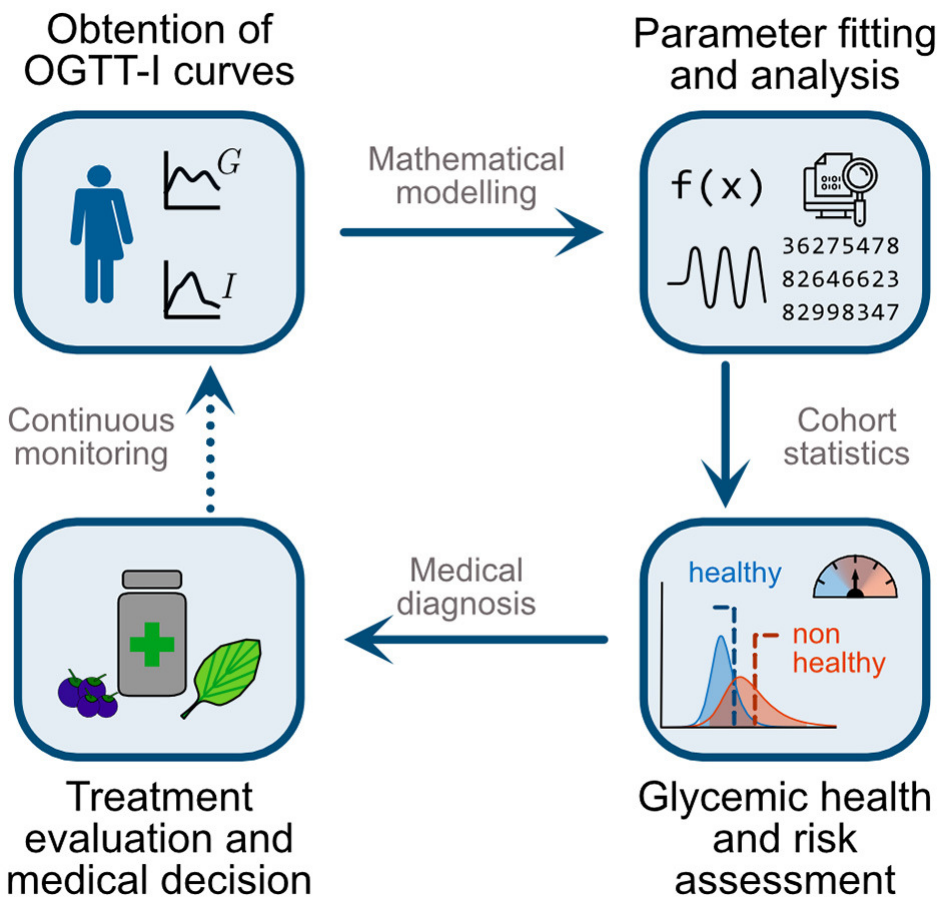
Schematic representation of the proposed methodology for a patient-wise assessment of nutritional and general interventions to reduce dysglycemia risk. First, individuals undergo an OGTT with five measurements for glycemia and insulinemia (5-point OGTT-I). Second, use the mathematical model and parameter fitting strategy of Contreras et al. (23) to obtain physiologically meaningful indexes to assess both the function and coordination among subsystems. Third, contrast individual values with those obtained in a cohort study. Here we evaluate whether a patient belongs to the healthy or non-healthy ranges and her/his overall risk of progressing to dysglycemic states. Fourth, using this assessment, practitioners may select suitable treatments or interventions, such as polyphenol-rich diets or supplementation, for targeted alteration of particular physiological subsystems and parameters. Finally, repeating the OGTT-I and monitoring over time enables the assessment and dynamic adaptation of interventions, for example adjustment of diet and polyphenolic supplementation targeting different subsystems according to the individual patient's response.

We organise our paper and its contributions as follows: First, we show that G-I health status encompasses a “healthy/non-healthy” continuum and that current binary diagnostic criteria would fail to identify individuals with a high risk of dysglycemia. We then use the mathematical model and parameter-fitting strategy in Contreras et al. (23) to obtain physiological values characterising the G-I status of a Chilean cohort (*n* = 1198) and provide a parameter-based definition of glycemic health. Then, using non-dimensional analysis, we assess the coordination between subsystems and explore how it varies between healthy and non-healthy individuals. Finally, we propose a statistical model to quantify the risk of dysglycemia given a single OGTT-I curve. Altogether, the method here proposed encompasses four steps, as described in Figure 2: (1). Individuals shall undergo a 5-point OGTT. (2). Using their curves and the model of Contreras et al. (23), we obtain their physiological parameters and non-dimensional numbers. (3). Using the cohort-derived values for parameter ranges, we assess the health status of each subsystem separately. (4). Using the statistical model, we assess the effectiveness of the treatment on reducing the risk of dysglycemia. As a proof of concept of this strategy, we followed individuals undertaking a polyphenol supplemented diet to show how to obtain early indicators of the dietary supplementation effects over glycemic control. Altogether, we provide evidence that this method is suitable to assess and follow nutritional interventions since it can detect subtle changes in the G-I subsystems, which otherwise would require invasive testing to be spotted.

## 2. Materials and Methods

### 2.1. Description of the Cohorts and Laboratory Analyses

In the observational study, we analysed individuals of both sexes, aged 18 to 65 years, undergoing a first-time 5-point OGTT analysis at Mutual de Seguridad (Santiago, Chile) or Fundación Médica Clínica San Cristóbal (Santiago, Chile), as part of a routine endocrino-metabolic evaluation ordered by their physicians. Exclusion criteria were a previous prediabetes or diabetes diagnosis or medically declared insulin resistance, chronic pharmacological therapy, body mass exceeding 30 kg/m^2^, lactating or pregnant women, cigarette smokers, or individuals with a history of drug or alcohol abuse. Medications affecting hepatic activity 28 days prior to the study, known allergies to medications, renal insufficiency and prediagnosed food allergies were also exclusion criteria.

We analysed the 5-point OGTT-I glycemia-insulinemia curves of selected individuals (*n* = 1198), with a median age of 33 (95% CI: [18, 64]). For OGTT, blood samples were taken at fasting level (basal, overnight) and every 30 min, for 2 h after the ingestion of 75 g glucose in 296 mL liquid (Trutol, Thermoscientific, Waltham, MA, USA.), consumed within less than 5 min. During the test, individuals were only allowed to ingest water. The analysis techniques for blood glucose and insulin were GOD-PAP colourimetry and chemiluminescence, respectively.

Individuals were classified as healthy if: (i) fasting glycemia and insulinemia are lower than 100 mg/dL and 15 μU/mL, respectively; (ii) glycemia values do not exceed 160 mg/dL and do not persist above 140 mg/dL after 2 h; (iii) insulinemia values do not exceed 100 μU/mL and do not persist above 60 μU/mL after 2 h. Consequently, the initial cohort was divided into groups of clinically “healthy” (*n* = 407) and “non-healthy” (*n* = 791) individuals. Individuals among the “healthy” cohort had a basal glycemia of 86.4 mg/dL (95% CI: [73.2, 97.2]) and a basal insulinemia of 4.32 μU/mL (95% CI: [2.02, 11.76]). Individuals among the “non-healthy” cohort had a basal glycemia of 91.0 mg/dL (95% CI: [75.3, 115.0]) and a basal insulinemia of 8.30 μU/mL (95% CI: [2.00, 29.02]). Approximately 80% of the cohort were female individuals, with ages ranging between 18 and 65 years. Nevertheless, no statistically significant sex-related differences were found (further details in Supplementary Figures S1, S4, S5, Supplementary Tables S2, S3, S6, S7).

For the case study on the effects of Delphinol® on the G-I dynamics, we studied OGTT-I curves of a group of individuals showing early symptoms of impaired G-I regulation, defined as having (i) fasting glycemia or insulinemia higher than 100 mg/dL and 15 μU/mL, respectively, or (ii) glycemia values exceeding 160 mg/dL or persisting above 140 mg/dL after 2 h; or (iii) insulinemia values exceeding 100 μU/mL or persisting above 60 μU/mL after 2 h. Under these criteria, all subjects would have been classified as non-healthy using the general cohort classification criteria. We screened for individuals aged 18 to 50 years, not having been previously diagnosed with prediabetes or diabetes, and with a body mass index (BMI) lower than 35 kg/m^2^ and higher than 25 kg/m^2^. Exclusion criteria were defined as fasting glucose higher than 126 mg/dL, use of hypoglycemic medication, current or past use of fibrates or statins during the past three months, lactating or pregnant women, cigarette smokers, or individuals with a history of drug or alcohol abuse or following any special diet. As with the general cohort, medications affecting hepatic activity 28 days prior to the study, known allergies to medications, renal insufficiency and prediagnosed food allergies were also used as exclusion criteria.

Clinical examination of subjects was performed at trial start and in weekly intervals until completion, measuring weight, height, waist circumference (at midpoint between the lowest rib and the iliac crest). Subjects were advised to avoid eating large food portions, especially carbohydrates, and refrain from strenuous exercise on days prior to their visit to the clinic. Volunteers were instructed to refrain from taking supplements including vitamins, not to change dietary or exercise habits, beginning from 7 days prior to participation in the trial. Acute non-prescription medications were permitted, but were required to be reported. Study participants were instructed to turn up at the clinic in the early morning (8 AM) having fasted overnight, at least 8 h, previous to each appointment. At this occasion fasting blood was drawn for subsequent analysis of basal blood glucose and insulin, followed by a 5-point OGTT as described previously.

The study was planned to comprise four different appointments for each subject with a period of one week between appointments, with an OGTT performed on each occasion. The first appointment consisted on an evaluation following the protocol described above, to determine control conditions with no treatment. A glass of 100 mL water (but no capsule) was given to each subject 15 min previous to this first OGTT in order to replicate the conditions of any given polyphenol dose in the next appointments. In each of the following appointments, subjects were given each time one different pre-prandial dose of Delphinol® (60, 120, and 180 mg), in randomised order, to be consumed with 100 ml of water 15 min before an OGTT. Neither the subjects nor the healthcare personnel taking the OGTT samples and measurements were aware of the dose contained inside each capsule. These dose levels were selected based on clinical results from Hidalgo et al. (24), who used a single 200 mg dose of Delphinol® in 10 volunteers, in order to investigate the effect of lower pre-prandial doses. Taking into account the size effect reported by these researchers on OGTT curves, we decided to use a 3.5-larger cohort to assess the effect of lower doses using the method proposed in this study. We used a commercially available Delphinol® food supplement from MNL-Group, Chile, a standardised maqui berry extract with a minimum content of 25% delphinidins and 35% total anthocyanins (polyphenolic composition in Supplementary Table S18), batch 13156, manufactured in 2013 by Anklam Extrakt, Germany, and encapsulated by Barnafi Krause Farmaceutica S.A., Chile. Capsules contained Delphinol bearing 31.5% delphinidin glycosides and 39.4% total anthocyanins (HPLC). All volunteers (*n* = 36) gave their informed consent to use their non-identifiable and confidential OGTT data in this study (Ethical Committee of Mutual de Seguridad, Santiago, Chile, resolution case #78).

### 2.2. Mathematical Model for the G-I Dynamics

We used a mathematical model to represent continuously the observed OGTT G-I curves (23). The model considers five compartments for describing G-I dynamics through coordinated subsystems coupled *via* parameters with physiological meaning, as represented in Figure 1E. Such compartments represent the amount (or concentration) of glucose in different compartments: in the stomach S, in the upper intestinal tract J (jejunum) and L (ileum), and in the bloodstream (glycemia) G. The last variable accounts for the insulinemia I. All magnitudes involved in the model are expressed in SI units; conversion factors for glycemia-related variables are 180 mg/dL = 10 mM, while for insulinemia 1 μU/mL = 6.945 pM.

To analyse the collective contribution groups of parameters involved in the G-I dynamics we used non-dimensional analysis (25–27). The number of independent Π numbers depends on the physical dimensions involved and the number of model parameters. The chosen scale variables were the basal levels of glycemia and insulinemia, the amount of glucose ingested, and an internal timescale that accounts for the ratio of incretion influence on pancreatic glucose perception versus the specific rate of glucose appearance in the bloodstream due to intestinal absorption. A complete mathematical description of the model non-dimensionalisation procedure is provided in the Supplementary Material, Section 4.

Statistical analysis and parameter-fitting were performed in MATLAB version R2020a, using the Global Optimization and Statistics toolboxes, partially executed on the Chilean National Laboratory for High-Performance Computation (NLHPC) servers. Other exploratory and statistical analyses were performed using the DMAKit-Lib Python library (28). Model equations and parameters/NDNs are described in the Supplementary Material, Section 4.

### 2.3. Statistical Analysis of Parameters/NDNs

**Figure 3:**
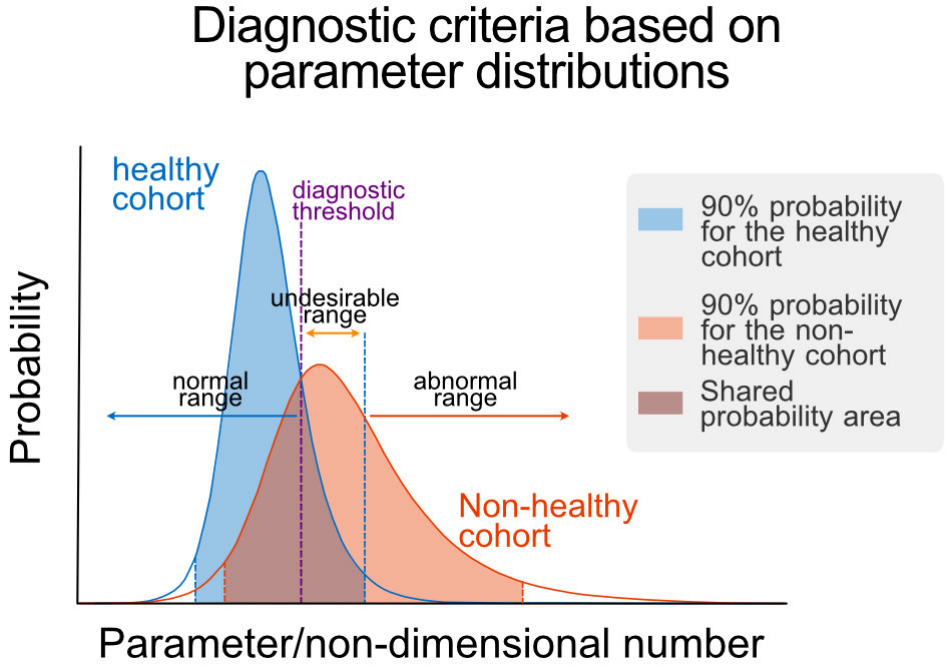
Parametric definition of health. Schematic representation of the determination of normal, undesirable, and abnormal ranges for a given parameter/NDN. These ranges are defined using the parameter/NDN distributions of the healthy and non-healthy cohorts, using the central 90% confidence interval of the healthy cohort distribution as limit of the non-healthy range and the point of equal probability density as the threshold between normal and undesirable ranges. Threshold values are reported in Table 1.

After obtaining parameters/NDNs for each individual, we obtained histograms of parameters/NDNs and assessed their distribution and ranges in healthy and non-healthy cohorts using log-logistic distributions. We then assessed differences in parameter/NDN distributions between cohorts using a Kolmogorov-Smirnov test. We determined the ranges of 90% left and right tails of the healthy population and compared the percentage of subjects in these ranges between healthy and non-healthy cohorts using a t-test. Significant differences were used to demonstrated shift towards the analysed tails of the distribution in non-healthy. We used the assigned shift trends to colour rows associated to each tail in Supplementary Tables S14, S15. To determine the healthy (“normal”) ranges for each parameter/NDN, we calculated the central 90% confidence interval of the healthy cohort distribution. Abnormal and undesirable ranges were determined as described in the text and Figure 3.

**Figure 4:**
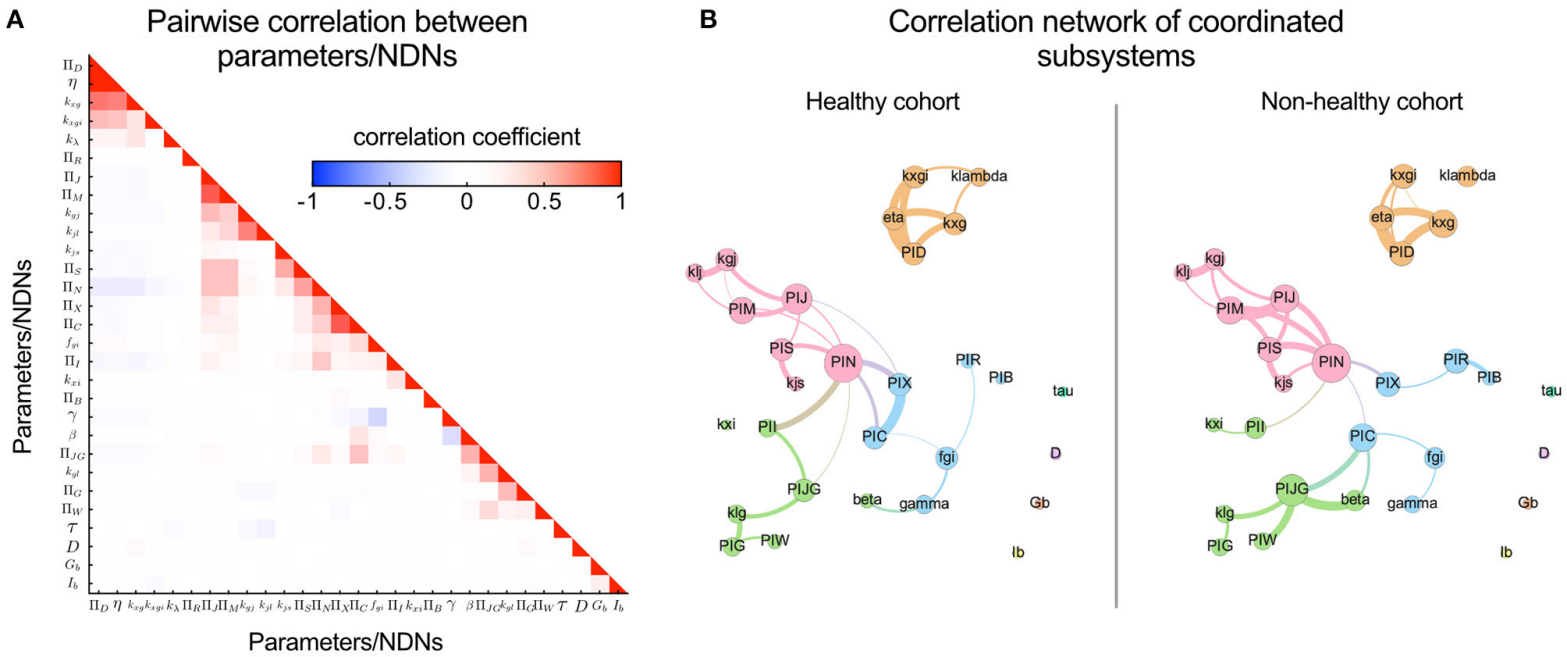
Pairwise correlation visualisation reveals covariating parameters/NDNs and local coordination between subsystems mediated by the incretin potency NDN (Π_*N*_). Coordination or independence between parameters can be quantified through their pairwise correlation coefficients; The higher the independence between Parameter/NDN, the lower their correlation coefficient. **(A)** Heatmap representing pairwise correlation between all model parameters/NDNs obtained for the general cohort. Cells are coloured according to the value of the pairwise correlation coefficient. The low colour saturation in cells indicates a general absence of redundancy between parameters/NDNs. **(B)** Visualising the correlation between parameters/NDNs in graph representations reveals communities of coordinated subsystems (represented in different colours). Each graph node (coloured circles) represents one parameter/NDN, and the node size is proportional to the number of other parameters/NDNs that are correlated to the node's parameter/NDN. Graph edges connect correlated parameters/NDNs, and their width is proportional to the absolute value of the correlation coefficient between connected nodes. Independent parameters have no connections between them. Three of these parameters/NDNs communities (pink, green, cyan) are loosely associated, mainly through the central Π_*N*_ node, while the fourth community is completely independent from the others (orange). Four parameters are non-correlated, including fasting glycemia and insulinemia, which remarks that basal levels do not directly reflect the health of any internal physiological system by themselves. Parameters/NDNs are more correlated in the non-healthy state, reflecting the loss of degrees of freedom and the strain to achieve glycemic control in non-healthy individuals.

Pairwise correlation coefficients were calculated for each parameter pair, collected into a correlation matrix, and used to generate graph representations of correlated groups of parameters/NDNs using the software GePhi, considering only those with absolute correlation coefficients larger than 0.4 (Figure 4B). Louvain's algorithm was applied to the structure of graphs to identify groups of highly correlated parameters, which were plotted and coloured differently in Figure 4B.

### 2.4. Sensitivity Analysis of Fitted Parameters

Considering that the parameter-fitting algorithm of Contreras et al. (23) involved heuristic steps, the fitting procedure was repeated at least five times for each patient in order to check the accuracy and uniqueness of parameter values in a particular range. Additionally, for each patient, a sensitivity analysis was performed in which the ten experimental G-I measures were varied up and down by 10% of its original value. This allowed us to determine parameters that were sensitive to errors in measurements and those that remained majorly unaffected. Conversely, this served to determine which parameters were well-defined by experimental points, and thus invariant, and which could be more variable for each patient.

### 2.5. ROC Curve Analysis and Optimal Classification Thresholds

Receiver operating characteristic (ROC) curves were built for each parameter/NDN, using separately the empirical histograms and the fitted distributions. We then derived a cut-off value for each parameter/NDN in both cohorts. For this, we calculated the optimal thresholds for classification of parameter/NDN values as healthy or non-healthy based on the maximum Youden's J index (Supplementary Table S12). Subsequently, the value of each individual's fitted parameter was used to classify the individual as healthy or non-healthy, depending on the relative position of the parameter value above or below the threshold.

### 2.6. Logistic Models for Dysglycemia Risk Probability

We constructed logistic regression models to determine which parameters/NDNs most influence clinical classification. To determine which parameter/NDNs are related to non-healthy states, we performed a Wald test, testing whether the relevant logistic regression coefficient was zero (null hypothesis) or different from it (alternative hypothesis). The test statistic (the estimated logistic regression coefficient divided by its standard error) follows the Standard Normal distribution, and its square follows the chi-squared distribution with 1 degree of freedom. To select the best combination of explanatory variables, we iteratively eliminated the parameter/NDN with the largest *p*-value in the Wald test for its coefficient in the regression model, continuing until all remaining coefficients were significant (*p* <0.05) in the Wald test. We built a full model with all parameters and NDNs and their pairwise interactions as predictors, and derived a simple model by eliminating all coefficients with non-significant *p*-values in the Wald test as described. We also constructed a quadratic model with all parameters and NDNs and their squared values as predictors and eliminated all non-significant parameters in a similar fashion, to obtain a quadratic model. Several model performance indexes were calculated and tabulated (Supplementary Table S12) to characterise the obtained models. Finally, we used the Matthews correlation coefficient (MCC) to assess the overall quality of the binary classification and the Akaike information criterion (AIC) to select the most appropriate model.

## 3. Results

### 3.1. OGTT Curves Form a Continuum Between Healthy and Non-healthy States

Current clinical criteria classify individuals as healthy or non-healthy based on threshold values for their OGTT responses. However, this classification disregards “healthy” individuals with a higher risk or tendency towards dysglycemia, who are those individuals where a diet rich in or supplemented with polyphenols may be the most suitable measure to avoid progression into a disease state. To illustrate how blurry this boundary is, we plotted glycemia and insulinemia OGTT curves from two cohorts classified as “healthy” or “non-healthy” according to current clinical criteria of normality (Figures 1A–D, respectively). The OGTT G-I curves and their interrelation describe how glycemic homeostasis is achieved by the physiological control system in each patient.

We observe a continuum of curves among individuals of the same cohort, suggesting that cut-off threshold values fail to describe other physiologically distinct states. Furthermore, we observe that glycemia and insulinemia profiles of the same individual are not always correlated; several possible shapes of insulinemia curves are possible for a given glycemia curve, and vice versa (cf. different colours in Figures 1A–D). These data suggest that G-I dynamic control is achieved through different mechanisms, supporting the idea that vulnerability to dysglycemia depends on individual circumstances and individual physiology. Besides, current binary diagnostic criteria fail to detect individuals at high risk of dysglycemia, who, however, would profit the most from dietary interventions/supplementation.

### 3.2. Parameter Sensitivity Shed Light on Predominant Mechanisms to Achieve Glycemic Control

To provide statistical grounds to our methodology, we first obtained physiological parameters governing each subsystem for all individuals in both cohorts mentioned above (cf. Figure 1E and Supplementary Table S13) using their 5-point OGTT-I curves. For this, we used the parameter-fitting strategy described in Contreras et al. (23). Next, to determine to what extent any parameter defines the shape of individual OGTT G-I curves, we performed a parameter sensitivity analysis for each individual in the healthy or non-healthy cohorts. Parameter sensitivity is a measure that allows quantifying how perturbations in the value of a parameter (i.e., physiological changes) affect the shape of the modelled OGTT curves. We classified “sensitive” and “insensitive” parameters based on whether they affect the height and shape of OGTT curve profiles in a patient-wise manner. Thus, sensitivity reflects how parameters influence G-I curve shapes for each individual or, in physiological terms, which subsystems are mainly responsible for the glycemic response in a given subject.

Furthermore, parameter influence on the shape of G-I curves may depend on an individual's health status rather than being general for all subjects. To assess whether a parameter's relative influence is affected by the G-I status, we compared parameter sensitivities in healthy and non-healthy cohorts. This is necessary for determining putative differences in the management of glycemic control from a quantitative perspective between healthy and non-healthy subjects. For this, we calculated the percentage of individuals for which each parameter is sensitive in the healthy and non-healthy cohorts separately. The relative contribution of a parameter to the shape of the curve may be compared when assessing percentages within and between cohorts (Supplementary Table S17) and, hence, for any individual.

Shifts in curve-shaping parameters, representing inter-individual differences in the management of glycemic control, can be found when comparing the healthy and non-healthy groups, although some parameters remain equally sensitive in both cohorts (*k*_*js*_, *f*_*gi*_, η, *D*, *k*_λ_, and γ), meaning that health status does not affect their related physiological subsystems differentially. Non-healthy subjects show a general decrease in parameter sensitivity (Supplementary Table S17).

Altogether, the data suggest non-healthy individuals over-rely on fewer subsystems that, in consequence, become more determinant to achieve G-I homeostasis. In physiological terms, this means that glycemic control in non-healthy individuals is less robust and prone to failure, while the function of some subsystems become less reliable. By contrast, healthy individuals have several subsystems that collectively contribute to determining OGTT curve shapes. For example, we observe that individuals currently classified as non-healthy show a more sensitive basal glucose consumption (*k*_*xg*_) and less sensitive insulin-dependent glucose consumption (*k*_*xgi*_) (Supplementary Table S17), suggesting a higher reliance on insulin-independent glucose consumption than in individuals classified as healthy. These observations are hallmarks of insulin resistance mechanisms (16). This also implies that dysglycemic individuals are likely to be less responsive to interactions between glycemia and insulinemia, thus reflecting the loss of glycemic control.

### 3.3. Quantifying Physiological Subsystem's Function and Defining Health From a Parametric Perspective

To evaluate each subsystem's relative influence on individual OGTT curves, we transformed the governing system of differential equations into their non-dimensional form and obtained independent non-dimensional Π numbers (NDNs). NDNs are meaningful combinations of physiologically consistent parameters and thus characterise different subsystems and help compare individuals on a parameter-independent scale (Figure 1E). All NDNs and the subsystems they represent are described and defined in Supplementary Tables S13, S14. We calculated pairwise correlations between all model parameters and NDNs among cohorts to assess mutual dependencies. Only a few showed significant correlations. Hence, the information contained in parameters/NDNs is not redundant (Figure 4A). Altogether, this analysis confirms that G-I dynamics can be represented in separate modular subsystems characterised by independent parameters/NDNs. Furthermore, parameters/NDNs dependencies are conserved for all subjects independently of their G-I status since correlation coefficients shown in Figure 4A are not significantly different between healthy and non-healthy groups (*p* > 0.05).

The next stage was to determine the health status of each subsystem involved in the G-I dynamics using Parameters/NDNs. We defined parameter/NDN normality ranges using cohort values, i.e., the distribution of parameter/NDN values between healthy and non-healthy cohorts (Figure 3). We characterised parameters/NDNs distributions using log-logistic distributions and calculated their 90% confidence intervals. We then selected parameters/NDNs with significantly different distributions between the healthy and non-healthy cohorts (highlighted in Supplementary Tables S15, S16) and defined thresholds to separate these cohorts in the parameter/NDNs space. The set of parameters/NDNs that differ between cohorts consists of *k*_*js*_, τ, *k*_*lg*_, η, *k*_*xgi*_, Π_*S*_, Π_*N*_, Π_*D*_, Π_*B*_, Π_*X*_, Π_*R*_, and Π_*I*_ (see Table 1). Thus, those parameters/NDNs can be used to assess any given individual's health status at the subsystem level.

**Table 1:** Healthy, undesirable and abnormal diagnostic ranges for model physiological parameters/NDNs.

**Physiological process**	**Variable**	**Units**	** Healthy diagnostic range **	** Undesirable diagnostic range **	** Abnormal diagnostic range **
	*k*_*js*_	min^−1^	0.0198 – 0.244	0.01 – 0.0198	<0.01
	τ	min	72 – 111	45 – 72	<45
**Digestive** **motility**	Π_*S*_	–	0.396 – 47.6	0.0013 – 0.396	<0.0013
**Glucose** **absorption**	*k*_*lg*_	min^−1^	0.00371 – 0.602	0.602 – 1.62	>1.62
**Incretin** **potency**	Π_*N*_	–	0.232 – 3.84	8.12 ×10^−4^ – 0.232	<8.12 ×10^−4^
	η	L^−3^	0.0145 – 0.178	0.178 – 6.97	>6.97
**Glucose** **distribution**	Π_*D*_	–	1.20 – 15.4	15.4 – 550	>550
	*k*_*xgi*_	min^−1^ pM^−1^	9.41 ×10^−6^ – 0.00259	5.67 ×10^−9^ – 9.41 ×10^−6^	<5.67 ×10^−9^
	Π_*B*_	–	0.00204 – 159	6.42 ×10^−7^ – 0.00204	<6.42 ×10^−7^
**Tisular** **glucose uptake**	Π_*X*_	–	0.00866 – 0.395	3.59 ×10^−7^ – 0.00866	<3.59 ×10^−7^
**Pancreatic** **response**	Π_*I*_	–	0.394 – 19.0	0.00871 – 0.394	<0.00871
**Hepatic** **response**	Π_*R*_	–	0.00933 – 41.0	41.0 – 134	>134
	*G*_*b*_	mM	4.10 – 5.03	5.03 – 5.29	>5.29
**Effective** **control levels**	*I*_*b*_	pM	13.1 – 47.9	47.9 – 68.6	>68.6

We used receiver-operator curves (ROCs) built for each parameter/NDN to find a diagnostic threshold that optimally separates healthy and non-healthy parameter values (Figure 3). These diagnostic thresholds closely coincided with the intersection between the healthy and non-healthy distribution curves. With these values, we defined three diagnostic ranges for each parameter/NDN: normal (from the threshold to the healthy side), undesirable (from the threshold to the non-healthy side but still inside the healthy cohort distribution), and abnormal (Table 1). In this way, a person's intervention requirements can be determined and targeted to specific subsystems according to their health status, as per the ranges where their parameters belong. Furthermore, as this method provides a detailed picture of the physiological function, it can also reveal new effects or targets of different polyphenolic formulations or molecules.

As a side remark, the traditional threshold-based diagnostic criteria using 2-point OGTT does not identify a person's individual risk: when using only basal (fasting) glycemia (*G*_*b*_) and insulinemia (*I*_*b*_) as predictors of dysglycemic risk, we observe around 20% of individuals in the healthy cohorts have out-of-bounds *G*_*b*_ values. Further, 50 and 65% of non-healthy subjects were out of range for *G*_*b*_ and *I*_*b*_, respectively. When considering out-of-range *G*_*b*_ and *I*_*b*_ as classification criteria, only 32% of non-healthy subjects are identified as such, whereas considering altered *G*_*b*_ or altered *I*_*b*_ misidentifies 39% of healthy subjects and 22% of non-healthy subjects.

### 3.4. Coordination Among Subsystems Affects OGTT Curve Shape

Physiological coordination among G-I subsystems can quantify the effectiveness of dietary interventions and/or supplementation with polyphenols to correct dysglycemic conditions in cohort studies. Parameter/NDN coordination gauges the status of internal subsystems, such as digestive motility and incretin action, that otherwise should be assessed by invasive methods (29–31). We explored the coordination among subsystems in the healthy and non-healthy cohorts using community detection algorithms applied on correlation graphs (Figure 4B), where correlated groups represent functional coordination between subsystems. We identified four correlated parameter/NDN communities (coloured in Figure 4B). We found that basal glycemia and insulinemia are not correlated to any other parameter/NDN, so the clinical status of internal subsystems cannot be inferred from these basal levels alone derived from the current 2-point OGTT procedure of diagnostic.

In the healthy cohort, we detected strong correlations between parameters/NDNs related to digestive motility, glucose absorption, pancreatic insulin secretion, and glucose consumption (Figure 4B), all connected to a central incretin load (Π_*N*_) node. In agreement with the current paradigm (31–36), this suggests the incretin activity load number (Π_*N*_) coordinates motility and glucose absorption along the digestive tract. These correlations are more notorious in the non-healthy cohort (Figure 4B), reflecting a higher reliance on incretin-mediated physiological coordination to achieve glycemic homeostasis. Accordingly, differences in correlated parameter/NDN values indicate that the non-healthy state is characterised by a slowing down of stomach and jejunal motility and a decrease in incretin load and pancreatic insulin secretion. Such hallmarks of glycemic control dysregulation have been validated experimentally in the past (29–31, 37). According to this observation, personalised dietary interventions with pharmacologically active polyphenols that target and improve digestive motility, incretin load and pancreatic insulin secretion parameters would be promising to ameliorate dysglycemia and slow its progression to disease states in subjects where these parameters are decreased.

### 3.5. A Statistical Model to Assess Health Status and Risk of Dysglycemia

Using the parameters/NDNs selected in the previous section, glycemic health status can be characterised and quantified in a patient-wise manner. For a given individual, the integration of the different parameters/NDNs values may indicate individual risk to dysglycemia at any given time. Here, we built a predictor of dysglycemic risk integrating all relevant parameters/NDNs describing each subsystem into a logistic regression model that quantifies an individual's probability of dysglycemia:


(1) $$\begin{array}{l} z=a_{1}\eta +a_{2}\Pi_{X}+a_{3}G_{b}+a_{4}I_{b}+a_{5}\tau +a_{6}k_{xgi}+a_{0}, \end{array}$$

(2) $$\begin{array}{l} \mathbb{P}\left(\text{dysglycemia}\right)=\frac{e^{z}}{1+e^{z}}, \end{array}$$

$$\begin{array}{l} {\;a}_{1}=0.5310,\;a_{2}=0.1960,\;a_{3}=1.2799\\ a_{4}=0.0330,\;a_{5}=-0.0187,\;a_{6}=-798.2059,\\ {\;a}_{0}=-6.0103. \end{array}$$

This risk parameter includes basal (fasting) glycemia and insulinemia but also physiological parameters representing glucose distribution (η), digestive motility (τ), and insulin sensitivity (*k*_*xgi*_, Π_*X*_). Therefore, this risk parameter combines the main internal physiological processes that determine the result of glycemic control as found in this work, and also the variables resulting from that glycemic control.

We built an ROC curve to assess the performance of this risk model when used as a classifier to discriminate between healthy and non-healthy individuals, as detailed in the Methods section. Performance metrics with an optimal threshold probability and comparison to other models (full and quadratic) are summarised in Supplementary Table S12. The simple model presented above was selected according to its larger Akaike Information Criterion (AIC). Using this parameter/NDN-based risk model, we observe that 26% of healthy subjects are classified as high-risk, somewhat higher than using basal values alone (≈20%) to classify dysglycemic abnormalities. Further, 77% of non-healthy individuals are successfully identified, achieving a diagnostic odds ratio of 9.3 (77% sensitivity and 74% specificity).

One of the advantages of this risk model is its ability to return not only an expected binary classification result but also a fitted probability of belonging to the positive class (38). The model considers a probability threshold of 0.60 or higher to classify a subject into the non-healthy group. However, probabilities can vary above and below that value, and those variations could signal the improvement and impoverishment of glycemic health. Moreover, since the model and parameters take into account several internal homeostatic mechanisms, they can be sensitive enough to quantify health changes that are difficult to univocally assess using other tools.

Therefore, analysing a 5-point OGTT coupled with the present approach can identify the risk of dysregulations in G-I dynamics earlier and more sensitively than current tools. This evaluation integrates an individual's personal vulnerability to dysglycemia and thus can help early decision making in favour of dietary interventions or supplementation with polyphenolic compounds which regulate glycemia rather than pharmacological treatment of disease states.

### 3.6. Evaluation of the Effect of Treatment With a Polyphenolic Extract in the Study Cohort

**Figure 5:**
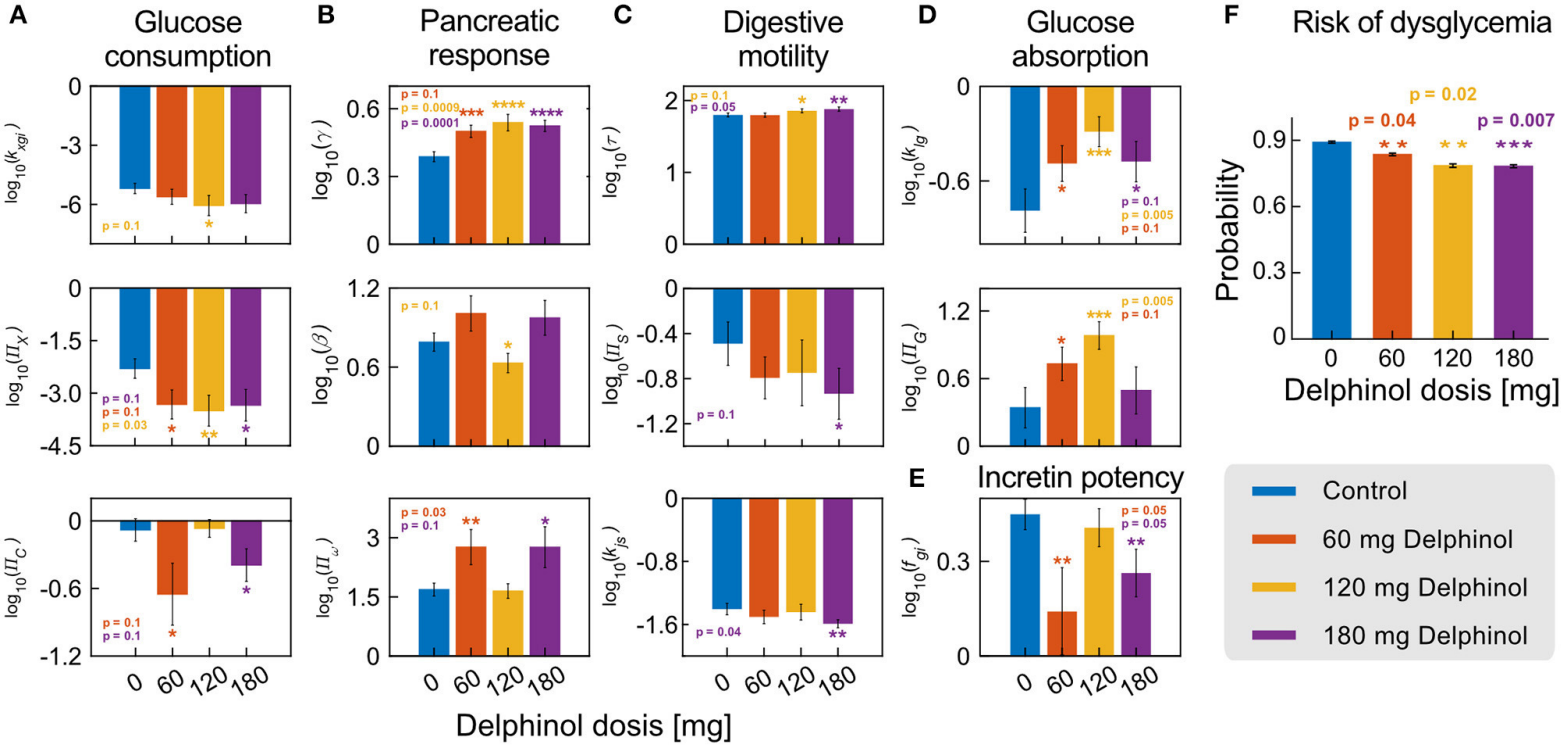
Delphinol® has a dose-dependent effect on different subsystems involved in G-I dynamics. **(A–E)** Comparison of the values of significantly varying parameters/NDNs between subjects in control conditions (no Delphinol®, blue bars) and treated with 60 mg (orange bars), 120 mg (yellow bars), and 180 mg (purple bars) Delphinol® prior to an OGTT. *p*-values are reported for the pairwise t-test comparison of the means of each treatment group with the control group. **(F)** Risk probabilities of dysglycemic (non-healthy) status calculated using the logistic model. The general risk of non-health status decreases with Delphinol® treatment in a dose-dependent way. Reported values are average probabilities with errors and *p*-values of the difference between each dose and the control group. In the figure, the number of asterisks denotes levels of significance of the difference with the control experiment; *p ≤ 0.1, **p ≤ 0.05, ***p ≤ 0.01, ****p ≤ 0.001.

To illustrate how individuals can be monitored during an intervention with a polyphenolic extract for dysglycemia treatment, we used Delphinol®, a standardised maqui berry (*Aristotelia chilensis*) extract that has been shown to improve glycemic control (39). We studied 36 hyperglycemic, non-diabetic, non-obese individuals randomly treated with three different pre-prandial doses of Delphinol® (control base with no treatment, 60, 120, and 180 mg), 15 min before an OGTT, with a one-week wash-out period between experiments. We used 5-points OGTT curves to calculate parameters/NDNs for each individual and Delphinol® dose. Statistically significant shifts in parameters/NDNs between the control and any treatment dose are highlighted in Figure 5.

Delphinol® supplementation induced significant changes (*p* < 0.05) towards the normality range defined for each parameters/NDNs. For example, the parameters/NDNs describing the digestive system's motility decrease (decreasing *k*_*js*_ and Π_*S*_; increasing τ) and absorption is delayed to the distal part of the small intestine. Interestingly, this target mechanism has also been described experimentally for metformin, one of the first-line drugs routinely used to treat hyperglycemic subjects (31–36), and also for flavonoids, anthocyanins and other polyphenols (6, 7, 10, 12, 18, 19, 40–42).

Using parameters/NDNs to describe subsystems we gained insights into the target mechanisms of action of Delphinol®. We observe the pancreatic response to incretin action (*f*_*gi*_) decreases, but the incretin activity load number Π_*N*_ remains the same. These data argue that digestive motility modulation occurs *via* a reduction of intestinal motility (τ) and the decreased action of incretins on the pancreatic function is enough to control glycemia reinforcing the idea that an improved glycemic control response is achieved by the coordinated participation of diverse mechanisms. Further, we observe that a single pre-prandial dose of Delphinol® can decrease dysglycemia risk from 89% (control) to 78% (180 mg dose), and that this effect occurs with any dose (Figure 5). Hence, Delphinol® is of particular interest for individuals with early-detected dysglycemia risk, for whom an intervention based on polyphenol rich food or supplementation is most desirable as an early intervention for dysglycemia or as a complement for traditional treatments to accelerate the restitution of homeostasis.

The results in Figure 5 that a single pre-prandial dose of Delphinol® significantly decreases the risk of dysglycemia in the studied cohort. Furthermore, results from significantly varying parameters and NDNs indicate that the general mechanisms of action for glycemic control improvement in the cohort include (a) decreasing the stomach emptying and intestinal transit rate, (b) delaying glucose absorption to the ileum, (c) improving pancreatic function, and (d) less dependency on the incretin effect for glycemic control. An additional paradoxical effect is observed, in which insulin-induced glucose consumption parameters (i.e., insulin sensitivity, quantified by *k*_*xgi*_ and Π_*X*_), decrease in a dose-dependent manner. As a proof of concept, in the next section we show the analysis of effects at the individual level.

We also show that some subsystems are more affected than others with the use of Delphinol®, suggesting the possibility that specific formulations might have specific target subsystems. This knowledge could lead to devising interventions with supplementation with Delphinol® combined with extracts with different polyphenolic molecules (e.g., other anthocyanins, flavonols, flavanols, and flavanones) affecting other subsystems, which could have synergistic effects to normalise all glycemic control physiological processes. For instance, cohort results show that single pre-prandial doses of Delphinol® does not seem to affect hepatic function in glucose management, and its effects on insulin sensitivity seems to be contrary to what should be expected. Therefore, we can expect any polyphenolic molecule or extract that ameliorates hepatic glucose management or insulin sensitivity to have synergistic effects when combined with single pre-prandial doses of Delphinol® as used in this study.

### 3.7. Patient-Wise Evaluation of the Effect of Treatment With a Polyphenolic Extract

**Figure 6:**
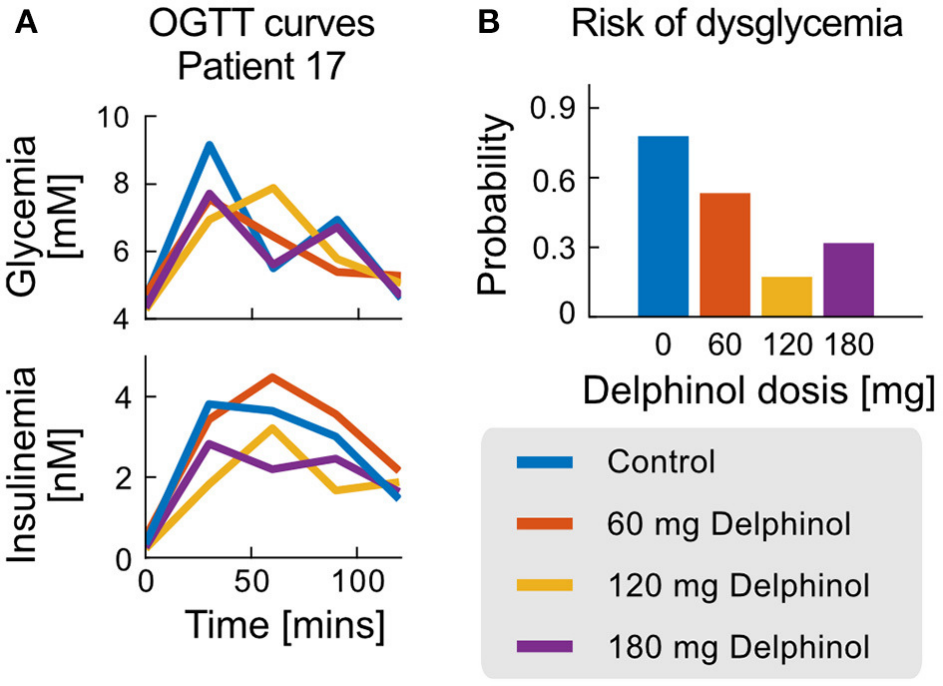
OGTT and probability of non-health status for the case of patient 17. **(A)** Glycemia and Insulinemia curves for the control experiment (no Delphinol®) and three preprandial doses (60, 120, and 180 mg). **(B)** Risk of non-health status calculated from the simple logistic model for patient 17 for the control experiment and the three different Delphinol® doses.

Dose-dependant effects of Delphinol® can be also analysed in a patient-wise manner. As an example, Figure 6 presents glycemia and insulinemia data for Patient 17, one of the subjects with most considerable parameter improvements with Delphinol® treatment. A diagnostic trend is not evident from the analysis of the crude OGTT curve values, and quantification of effects is even less possible from the raw curves alone.

In general, the use of the dysglycemia risk test as a diagnostic tool demonstrates a general improvement of the glycemic control response in patients treated with Delphinol® through non-obvious balancing mechanisms. In Patient 17, quantification of the degree of improvement in glycemic control using the dysglycemia risk test demonstrates the sensitivity of the method to detect slight changes in glycemic control efficacy, which are difficult to asses with other methods. This can be used together with the exact variations observed in each parameter/NDN to quantify the health status of each subsystem of the glycemic control system and their contribution to the non-healthy risk for each patient.

Parameters and NDNs can be used to characterise the function of the different subsystems that intervene in glycemic regulation during an OGTT in a patient-wise manner. The NDNs ranges shown in Table 1 allow assessing and comparing treatment efficacy in each patient. Figure 6 depicts the range of all physiological subsystem's characteristic NDN and basal glycemia and insulinemiafor each patient (classified as healthy, undesirable and abnormal), reflecting how pre-prandial Delphinol® consumption affects glycemic control physiological subsystems for a set of exemplary patients.

**Figure 7:**
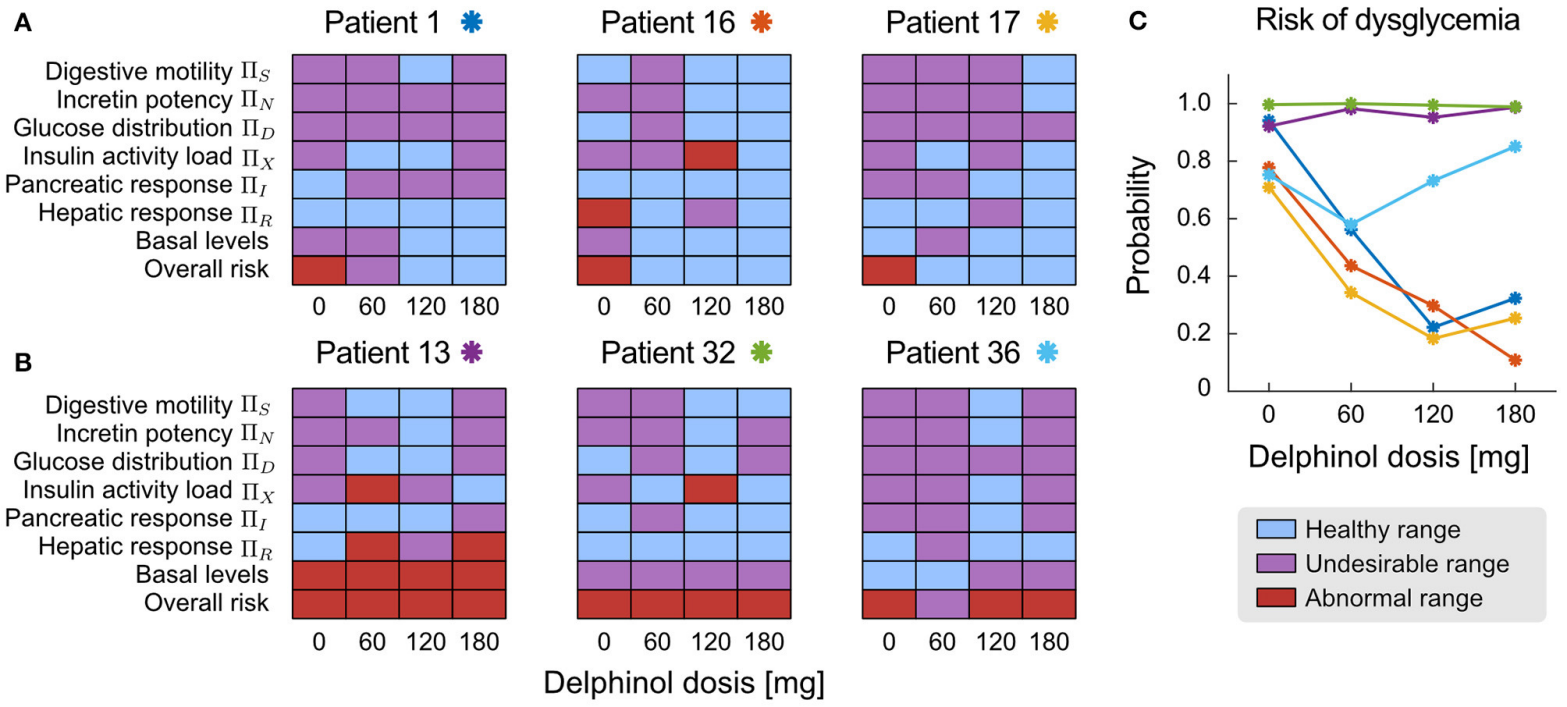
Patient-wise assessment of the dose-dependent effects of pre-prandial Delphinol®. In the general cohort, some individuals reacted more positively to Delphinol® than others, decreasing their dysglycemia risk. This figure presents exemplary results for both types of patients. **(A)** Patients 1, 16, and 17 show a clear and dose-dependent improvement of their overall glycemic function expressed by the normalization of NDNs related to physiological subsystems involved in glycemic control. **(B)** Patients 13, 32, and 36 do not clearly respond to pre-prandial Delphinol® treatment in the doses considered by this study, as assessed by the less pronounced effect on normalizing the NDNs of internal physiological subsystem. **(C)** Effect of pre-prandial Delphinol® on the overall risk of dysglycemia, yielding consistent results for the same patients.

Patients 1, 16, and 17 show a marked improvement in their dysglycemia risk (Figure 7C). Reflecting this risk decrease, Figure 7A shows that higher doses in all patients share an improvement in digestive motility, basal glycemia and insulinemia, and insulin activity load (patient adjusted proxy for insulin sensitivity). Incretin potency, glucose distribution, and pancreatic response are also improved, although less dose-dependent. The 180-mg dose is undoubtedly the most effective for Patient 16 and Patient 17. In contrast, Patient 1 seems to benefit the most with 120 mg of Delphinol® to normalise physiological parameters.

In contrast, treatment with pre-prandial Delphinol® has a minimal effect on reducing dysglycemia risk in Patients 13, 32, and 36 using the doses assayed in this study. Patient 36 is a special case since their basal glycemia and insulinemia are normal, but the overall risk of dysglycemia is high. Dysglycemia risk can be detected when assessing NDN values and signal physiological processes that are not fully functional. The Delphinol® treatment effects on physiological NDNs and basal levels are heterogeneous in these three patients and do not show a noticeable dose-dependent trend. However, all of them present a general normalisation of the function of internal physiological subsystems with the 120-mg dose. In view of these results, a healthcare practitioner could decide that (a) pre-prandial Delphinol® is not effective for these patients, so other polyphenolic treatments should be used, (b) a 120-mg pre-prandial Delphinol® dose should be used together with another synergistic intervention to decrease dysglycemia risk, and (c) larger pre-prandial Delphinol® doses or longer Delphinol® dosages should be assayed with these patients.

The paradoxical effect observed in the cohort analysis, in which insulin-induced glucose consumption parameters decrease in a dose-dependent manner, is clearly explained when separating individuals for which the treatment is effective (Figure 7A) and those for which no improvement in dysglycemia risk is observed (Figure 7B). For the most effective dose, insulin sensitivity is normalised along other physiological subsystems in patients whose dysglycemia risk decreases, while for those individuals for which the treatment does not decrease the risk, the most effective dose does not normalise insulin sensitivity. This reflects the relationship between risk and insulin sensitivity expressed in the equations defining the calculation of dysglycemia risk. Therefore, data show that only patients who respond to pre-prandial Delphinol® treatment improve their insulin sensitivity and dysglycemic risk, but not all individuals respond in this manner. Hence, the decrease in insulin sensitivity in the general cohort should reflect a more general unresponsiveness to pre-prandial Delphinol® to ameliorate insulin sensitivity, and Delphinol®'s general positive effect on dysglycemia risk may be more related to an improvement of other physiological subsystems, through the other action mechanisms presented above.

## 4. Discussion

In this work, we present evidence supporting the notion that dysglycemia risk can be masked by current threshold-based diagnostic criteria and that vulnerability to dysglycemia depends on individual circumstances and physiology. Such findings are in line with those of Hall et al. (43), who used continuous glucose monitoring to test such assumptions and found that individuals have specific patterns of post-meal glycemic responses, which reflect each subject's differential G-I vulnerability. This work and others (1, 2, 44) indicate that glucose dysregulation is more prevalent and heterogeneous than previously thought and can affect individuals considered normoglycemic by the current clinical criteria.

Here, we present a new strategy to assess individual glycemia health status in a non-invasive 5-point OGTT coupled to modelling. The method enables the early identification of individuals at risk of progressing to dysglycemic states. Once identified, nutritional interventions based on diets rich in or supplemented with polyphenols may be used as the first line of intervention to lower the risk cost-effectively. Further, since a growing body of evidence suggests several polyphenolic compounds can ameliorate type 2 diabetes patients (6–8, 16, 41, 42, 45, 46), the progression of the intervention may be followed by assessing the health status of each subsystem at different times and the possible reversion of the dysglycemic state may be assessed at any moment.

This approach builds on the knowledge obtained by analysing the healthy and non-healthy cohorts. Cohort statistics enabled us to (i) quantify the healthy function of the different subsystems involved in the G-I dynamics and (ii) know their coordination in healthy and non-healthy individuals. By having the cohort-derived normality ranges as a blueprint, we can assess new patients' dysglycemia risk and health status at the subsystem level and from a parametric point of view. Therefore, this parametric health assessment can serve to study complex questions. For example, whether all or which polyphenolic compounds ameliorate dysglycemia. This strategy may be used in the future to analyse which polyphenolic compounds claiming to be anti hyper-glycemic target which physiological subsystems or whether a known compound can affect other subsystems than those already reported. This study allowed us to identify the target subsystems of the commercial polyphenolic formulation Delphinol®. This supplement exerts its glycemic control properties by ameliorating digestive motility, fasting glycemia and insulinemia, insulin sensitivity. Secondary targets are incretin potency, glucose-distribution, and pancreatic function.

Until now, physiological parameters describing the subsystems involved in G-I dynamics, such as digestive motility rates, glucose absorption, pancreatic function, incretin activity, and hepatic reaction, could only be obtained by complex research-lab tests (29–31). Coordination among subsystems gives further insight on which functions are affected when progressing through dysglycemic states to disease. Furthermore, coordination allows a systematic measure in cohort studies of nutritional interventions, particularly those whose effects are subtle and time-dependent, such as polyphenol-rich diets.

To sum up, we present a patient-wise methodology to assess glycemic health status and dysglycemia risk, with applications in polyphenol research and nutritional interventions. This methodology allows assessing the effectiveness of such interventions by detecting subtle changes in the patient's 5-point OGTT profiles that current diagnostic criteria would neglect. The strategy enables continuous monitoring and detection of subtle changes (improvements or fall-outs) in the health status. In turn, this allows rapid medical decisions in a fairly straightforward manner. For example, through this strategy, health practitioners may rapidly adjust and tailor the intervention to individual needs to maximise the polyphenolic supplementation impact. Altogether, patient-wise comparison of the effects and combination of different interventions can pave the road to building a precision preventive medicine approach for glycemic dysregulation based on polyphenols and other dietary and non-dietary interventions.

## Data Availability Statement

The datasets analysed are available from the corresponding authors upon reasonable request.

## Ethics Statement

The studies involving human participants were reviewed and approved by Ethical Committee of Mutual de Seguridad, Santiago, Chile, resolution case #78. The patients/participants provided their written informed consent to participate in this study.

## Author Contributions

ÁO-N, SC, DM-O, PV, CC, and MT: conceptualisation. SC, DM-O, ÁO-N, and CC: methodology. ÁO-N and PV: data curation. ÁO-N, MT, AL, and CC: validation. ÁO-N, SC, and DM-O: investigation. ÁO-N, SC, DM-O, and MT: writing, original draft preparation. SC, ÁO-N, AL, and MT: writing, review, and editing. ÁO-N and CC: supervision. ÁO-N and SC: visualisation. ÁO-N and AL: project administration. ÁO-N, SC, and AL: funding resources.

## Funding

Open Access publication has been enabled by the Max-Planck-Society. SC received support from the Max-Planck-Society. DM-O gratefully acknowledges Conicyt, Chile, for Ph.D. fellowship 21181435. MT gratefully acknowledges Fondecyt Project 1191727. The authors gratefully acknowledge funding from the Centre for Biotechnology and Bioengineering—CeBiB (PIA project FB0001, Conicyt, Chile) and MNL-Group. The authors declare that this study received funding from MNL-Group. This funder was not involved in the study design, collection, analysis, interpretation of data, the writing of this article or the decision to submit it for publication.

## Conflict of Interest

AL is currently employed by MNL-Group, company producing Delphinol®. However, he did not participate in conceptualisation of the study, data collection, investigation, processing, or analysis of the results. The remaining authors declare that the research was conducted in the absence of any commercial or financial relationships that could be construed as a potential conflict of interest.

## Publisher's Note

All claims expressed in this article are solely those of the authors and do not necessarily represent those of their affiliated organizations, or those of the publisher, the editors and the reviewers. Any product that may be evaluated in this article, or claim that may be made by its manufacturer, is not guaranteed or endorsed by the publisher.
