# Supplementary material for: Patient-Wise Methodology to Assess Glycemic Health Status: Applications to Quantify the Efficacy and Physiological Targets of Polyphenols on Glycemic Control
Source: Front Nutr. 2022 Feb 17;9:831696. doi: 10.3389/fnut.2022.831696 (PMC8892255; doi:10.3389/fnut.2022.831696)
Supplement: Supplementary file 1 [file Data_Sheet_1.PDF]

Supplementary Material

1 DESCRIPTION OF THE WHOLE, HEALTHY AND NON-HEALTHY COHORTS

The dataset analyzed in this work comprises glycemia and insulinemia measures of 5-point OGTT tests ($n = 1198$). Blood samples were taken at fasting (basal, overnight) level and every 30 minutes, for 2 hours after the ingestion of 75 g glucose in 296 mL liquid (Trutol, Thermoscientific, Waltham, MA, USA.), consumed within less than 5 min (c.f. Materials and Methods section in the main manuscript). During the test, individuals were only allowed to ingest water. The analysis techniques for blood glucose and insulin were GOD-PAP colorimetry and chemoluminescence, respectively. Individuals were clinically classified according to the health criteria described in Materials and Methods in the main manuscript to characterize normoglycemic subjects based on their OGTT G-I profiles, further described in Tab S1. Consequently, the initial cohort was divided into groups of clinically "healthy" ($n = 407$) and "non-healthy" ($n = 791$) individuals.

Table S1. Health criteria used to classify subjects as normoglycemic based on their OGTT G-I profiles.

Criterion	Glycemia Criterion (mg/dL)	Insulinemia Criterion μ U/mL
Basal maximum	100	15
Two-hours value maximum	140	60
Any time maximum	160	100

1.1 Statistical descriptors of glycemia and insulinemia measurements in the healthy and non-healthy cohorts grouped by sex

Sex distribution within the healthy and non-healthy cohorts is presented in Fig S1.

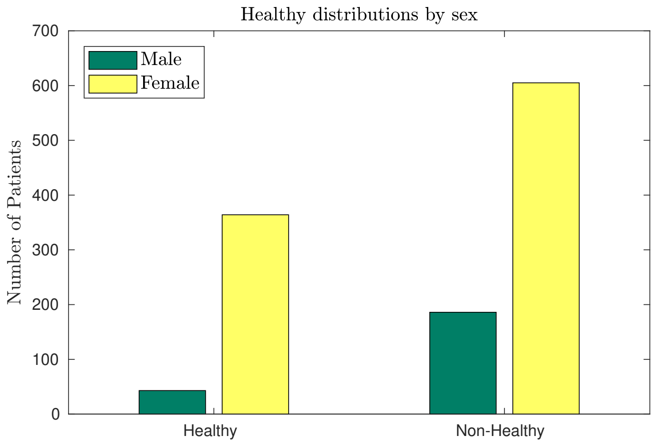

Figure S1. Sex distribution within healthy and non-healthy cohorts.

We calculated different statistical descriptors for glycemia and insulinemia measurements within healthy and non-healthy cohorts, and within male and female subjects. Values are summarized in Tabs S2, S3, S4, and S5.

Table S2. Statistical summary for glycemia and insulinemia time-points in male subjects)

Statistical summary for male subjects						
**Measure**	**Mean**	**Std Dev**	**Min**	**Max**	**Quartile 1**	**Quartile 3**
Basal glycemia	96.1	12.2	74.0	205.0	89.0	101.0
Basal insulinemia	11.2	7.5	2.0	47.9	5.3	15.1
Glycemia 30 min	156.3	31.4	78.0	306.0	136.0	175.0
Insulinemia 30 min	105.0	71.2	7.3	300.0	45.2	153.0
Glycemia 60 min	151.8	42.8	55.0	316.0	129.0	175.0
Insulinemia 60 min	108.1	71.7	6.7	300.0	51.3	155.0
Glycemia 90 min	132.5	38.5	55.0	309.0	104.0	159.0
Insulinemia 90 min	94.6	74.8	10.5	300.0	38.4	127.0
Glycemia 120 min	116.7	34.9	38.0	324.0	96.0	133.0
Insulinemia 120 min	80.0	73.2	4.6	300.0	31.0	100.0

Table S3. Statistical summary for glycemia and insulinemia time-points in female subjects

Statistical summary for female subjects						
**Measure**	**Mean**	**Std Dev**	**Min**	**Max**	**Quartile 1**	**Quartile 3**
Basal glycemia	88.6	9.6	9.0	142.0	82.0	94.0
Basal insulinemia	7.8	6.4	2.0	72.1	3.4	9.9
Glycemia 30 min	136.9	28.5	69.0	295.0	118.0	153.0
Insulinemia 30 min	79.9	67.5	2.0	846.0	37.3	99.0
Glycemia 60 min	131.8	40.0	11.0	421.0	104.0	156.0
Insulinemia 60 min	81.6	63.4	5.1	410.0	39.8	103.0
Glycemia 90 min	120.0	37.4	0.0	341.0	94.0	140.0
Insulinemia 90 min	72.9	59.5	0.0	381.0	34.4	86.7
Glycemia 120 min	113.6	32.3	46.0	321.0	92.0	131.0
Insulinemia 120 min	67.4	59.6	2.7	486.0	31.0	79.2

Table S4. Statistical summary for glycemia and insulinemia time-points in the healthy cohort

Statistical summary for healthy subjects						
**Measure**	**Mean**	**Std Dev**	**Min**	**Max**	**Quartile 1**	**Quartile 3**
Basal glycemia	85.6	6.6	63.0	100.0	81.0	90.0
Basal insulinemia	5.0	2.8	2.0	14.9	2.9	6.7
Glycemia 30 min	122.4	18.9	69.0	160.0	109.0	136.0
Insulinemia 30 min	45.4	22.3	2.0	100.0	28.2	59.7
Glycemia 60 min	111.9	24.0	54.0	160.0	95.0	130.0
Insulinemia 60 min	44.7	20.0	5.1	100.0	30.0	57.6
Glycemia 90 min	101.6	21.5	52.0	156.0	86.0	116.0
Insulinemia 90 min	38.4	17.2	6.5	98.6	26.4	47.7
Glycemia 120 min	97.3	18.5	38.0	140.0	86.0	109.0
Insulinemia 120 min	32.9	13.9	3.5	60.0	21.7	43.8

Figs S2, S3, S4, and S5 show box-plots of the data summarized in the previous tables for visual dispersion analysis.

Table S5. Statistical summary for glycemia and insulinemia time-points in the non-healthy cohort

Statistical summary for non-healthy subjects						
**Measure**	**Mean**	**Std Dev**	**Min**	**Max**	**Quartile 1**	**Quartile 3**
Basal glycemia	92.1	11.4	9.0	205.0	85.0	98.0
Basal insulinemia	10.2	7.5	2.0	72.1	5.0	13.1
Glycemia 30 min	149.8	30.3	69.0	306.0	127.0	167.0
Insulinemia 30 min	105.1	75.9	5.2	846.0	49.8	145.0
Glycemia 60 min	147.8	43.0	11.0	421.0	118.0	173.0
Insulinemia 60 min	108.4	70.7	9.3	410.0	52.9	146.0
Glycemia 90 min	133.3	40.1	0.0	341.0	104.0	158.0
Insulinemia 90 min	97.2	68.6	0.0	381.0	47.7	131.0
Glycemia 120 min	123.3	35.2	46.0	324.0	99.0	141.0
Insulinemia 120 min	89.4	68.9	2.7	486.0	42.0	111.0

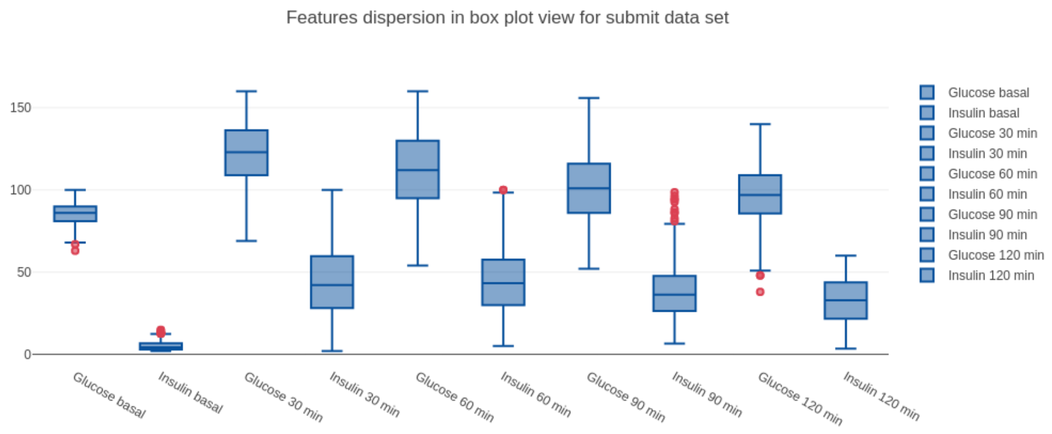

Figure S2. Dispersion analysis for the healthy cohort

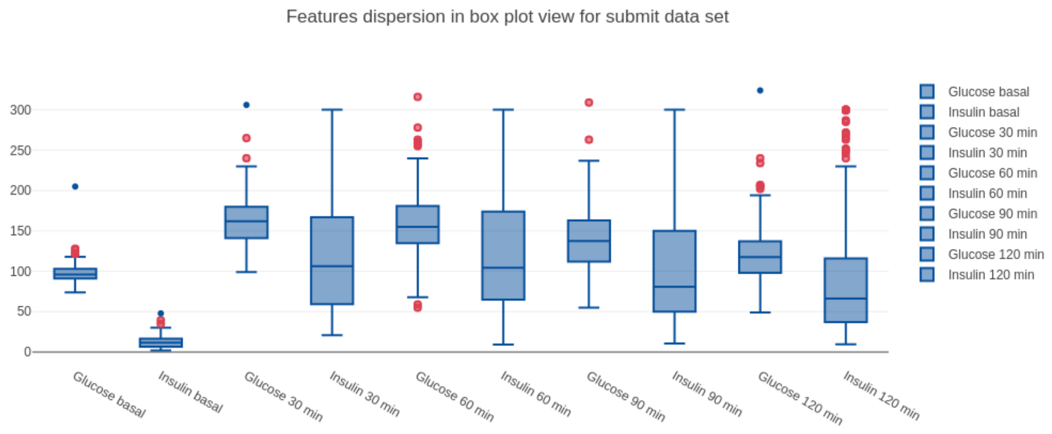

Figure S3. Dispersion analysis for the non-healthy cohort

1.2 Comparison and analysis of statistical distributions by sex

Sex-related dependency in the dataset was assessed by using a non-parametric test applied to the healthy and non-healthy cohorts. Each measure was assessed using the Mann-Whitney test (U-test). No statistically

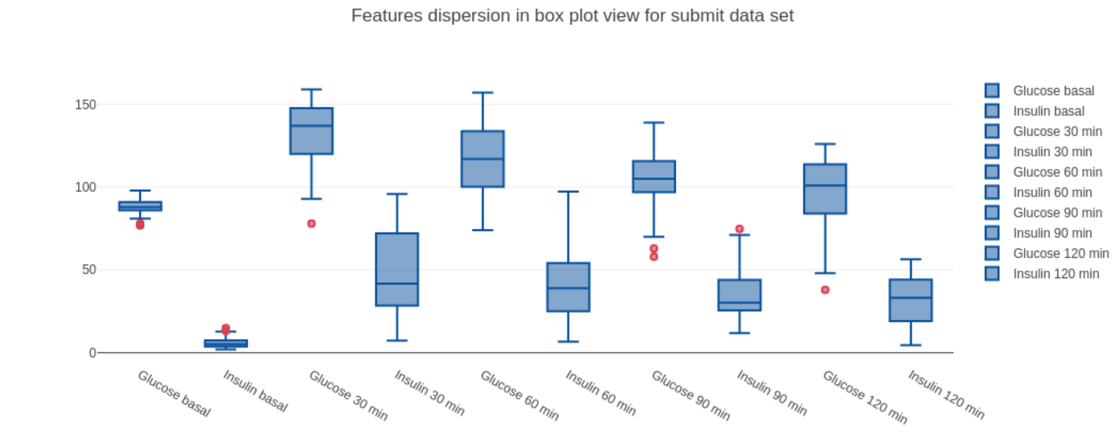

Figure S4. Dispersion analysis for male subjects

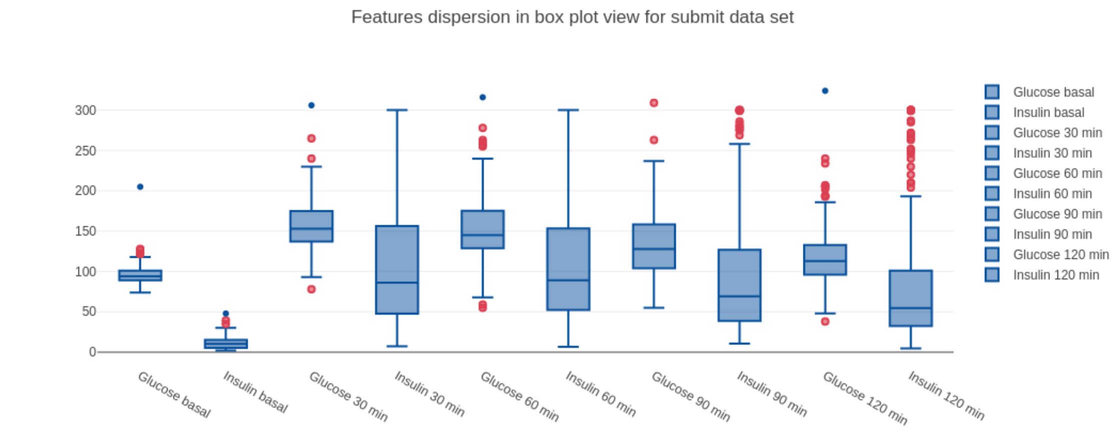

Figure S5. Dispersion analysis for female subjects

meaningful difference was found between male and female subjects in both cohorts. All results are summarized in Tables S6 and S7.

Table S6. Statistical assessment of sex-related differences in the healthy cohort

Mann-Whitney test results for sex-related differences in the healthy cohort			
**Measure**	**Statistic**	***p*-value**	**Result**
Basal glycemia	95494.5	0.2456	No difference
Basal insulinemia	96008.0	0.2895	No difference
Glycemia 30 min	94848.0	0.1956	No difference
Insulinemia 30 min	97480.0	0.4339	No difference
Glycemia 60 min	96740.5	0.3592	No difference
Insulinemia 60 min	97553.0	0.4414	No difference
Glycemia 90 min	97006.0	0.3855	No difference
Insulinemia 90 min	97085.5	0.3936	No difference
Glycemia 120 min	97542.0	0.4403	No difference
Insulinemia 120 min	97723.0	0.4591	No difference

Table S7. Statistical assessment of sex-related differences (non-healthy cohort)

Mann-Whitney test results for sex-related differences in the non-healthy cohort			
**Measure**	**Statistic**	***p*-value**	**Result**
Basal glycemia	38318.0	0.6792	No difference
Basal insulinemia	45473.5	0.1804	No difference
Glycemia 30 min	43758.0	0.4988	No difference
Insulinemia 30 min	52777.0	0.1000	No difference
Glycemia 60 min	49518.0	0.2509	No difference
Insulinemia 60 min	52092.0	0.3000	No difference
Glycemia 90 min	55650.5	0.3056	No difference
Insulinemia 90 min	56605.0	0.0131	No difference
Glycemia 120 min	60117.5	0.1441	No difference
Insulinemia 120 min	61261.5	0.2469	No difference

2 DATASETS FOR THE DELPHINOL® EFFECT STUDY

The different glycemia and insulinemia curve measurements for subjects in the Delphinol® case study dataset with different treatment dosages are shown jointly in Fig S6.

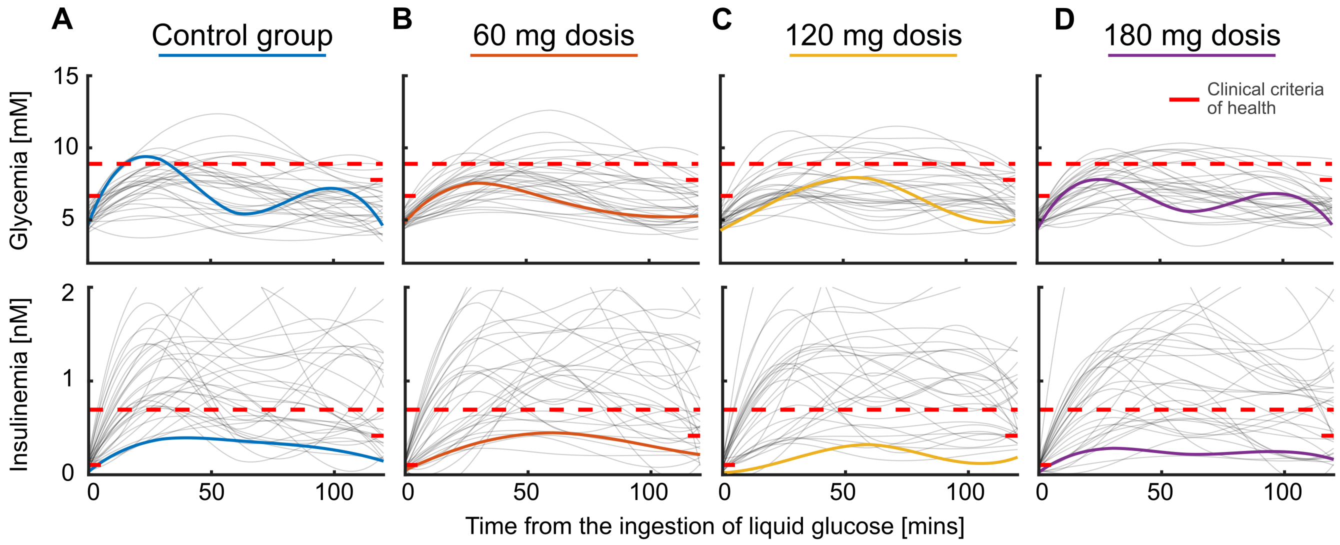

Figure S6. Glycemia and insulinemia curves for the Delphinol® effect study on G-I homeostasis. Subjects were treated with three doses of Delphinol® as described in the Materials and Methods section of the main manuscript. Results for the control experiment (no Delphinol®) and the 60 mg, 120 mg and 180 mg Delphinol® experiments are plotted on **A–D**, respectively.

Statistical summaries for glycemia and insulinemia measurements at each dosage are presented in Tables S8, S9, S10, and S11.

Table S8. Statistical summary of the results of the control experiment in the Delphinol® effect study

Statistical summary for the control experiment						
**Measure**	**Average**	**std**	**Min**	**Max**	**Quartile 1**	**Quartile 3**
Basal glycemia	91.7	9.8	74.0	117.0	85.00	96.50
Glycemia 30 min	139.6	27.9	69.0	199.0	127.25	159.25
Glycemia 60 min	126.8	33.7	71.0	220.0	101.25	143.75
Glycemia 90 min	114.8	27.9	56.0	164.0	95.75	127.75
Glycemia 120 min	105.9	26.5	63.0	161.0	88.00	124.25
Basal insulinemia	15.5	6.8	4.9	30.8	10.28	20.98
Insulinemia 30 min	148.1	62.4	54.9	315.0	104.25	194.00
Insulinemia 60 min	143.1	93.3	23.0	518.0	76.40	179.50
Insulinemia 90 min	117.1	70.2	15.0	312.0	62.85	169.0
Insulinemia 120 min	116.2	93.1	20.2	380.0	47.05	171.75

Table S9. Statistical summary of the results of the 60 mg dosage experiment in the Delphinol® effect study

Statistical summary for the 60 mg dosage experiment						
**Measure**	**Average**	**std**	**Min**	**Max**	**Quartile 1**	**Quartile 3**
Basal glycemia	88.9	7.1	77.0	103.0	83.00	93.00
Glycemia 30 min	136.0	21.2	99.0	188.0	117.75	147.75
Glycemia 60 min	139.1	28.6	92.0	227.0	121.50	154.75
Glycemia 90 min	121.6	29.9	74.0	186.0	97.00	143.50
Glycemia 120 min	110.8	24.9	66.0	171.0	95.00	126.75
Basal insulinemia	13.8	7.4	3.7	37.6	7.95	17.98
Insulinemia 30 min	138.0	81.3	34.7	323.0	69.57	209.25
Insulinemia 60 min	156.3	82.5	32.1	393.0	93.63	199.25
Insulinemia 90 min	143.0	81.8	17.6	300.0	74.40	204.25
Insulinemia 120 min	105.0	79.4	9.9	300.0	46.90	172.00

Table S10. Statistical summary of the results of the 120 mg dosage experiment in the Delphinol® effect study

Statistical summary for the 120 mg dosage experiment						
**Measure**	**Average**	**std**	**Min**	**Max**	**Quartile 1**	**Quartile 3**
Basal glycemia	88.5	7.9	77.0	112.0	83.75	92.00
Glycemia 30 min	132.9	25.7	53.0	201.0	122.75	146.25
Glycemia 60 min	134.1	32.5	75.0	201.0	111.00	156.25
Glycemia 90 min	126.1	30.1	74.0	194.0	104.00	148.00
Glycemia 120 min	111.4	21.9	80.0	152.0	94.00	128.50
Basal insulinemia	13.8	7.3	3.2	30.9	8.00	19.50
Insulinemia 30 min	140.5	76.8	26.2	371.0	76.23	185.00
Insulinemia 60 min	146.9	91.8	25.0	528.0	75.52	184.25
Insulinemia 90 min	148.0	119.0	11.8	706.0	68.45	190.25
Insulinemia 120 min	118.8	81.7	27.0	399.0	62.55	149.50

3 PERFORMANCE OF DIAGNOSTIC LOGISTIC MODELS FOR ASSESSING DYSGLYCEMIA RISK

In order to build predictors for the risk of dysglycemia, we used all model parameters and NDNs that were significantly different between healthy and non-healthy groups to construct a log-logistic regression model using all parameters (full model). We then performed a step-wise elimination of regression coefficients, removing the highest non-significant term until the model conserved only significant coefficients ($p < 0.05$, Wald test). The resulting simple model was presented in the main manuscript and used to analyze the results of the Delphinol® effect study. In order to test whether quadratic variables could be used to build

Table S11. Statistical summary of the results of the 60 mg dosage experiment in the Delphinol® effect study

Statistical summary for the 180 mg dosage experiment						
**Measure**	**Average**	**std**	**Min**	**Max**	**Quartile 1**	**Quartile 3**
Basal glycemia	88.1	7.5	71.0	104.0	83.00	92.25
Glycemia 30 min	134.0	24.6	86.2	179.0	116.25	152.50
Glycemia 60 min	129.1	28.5	59.0	186.0	112.50	143.25
Glycemia 90 min	119.4	24.8	79.0	175.0	101.50	137.25
Glycemia 120 min	105.4	17.8	68.0	166.0	96.00	114.00
Basal insulinemia	12.1	5.9	3.7	26.9	7.07	15.95
Insulinemia 30 min	136.6	87.1	17.0	477.0	74.68	185.00
Insulinemia 60 min	136.7	73.0	29.0	301.0	78.63	196.50
Insulinemia 90 min	117.4	71.4	22.2	310.0	60.85	163.00
Insulinemia 120 min	100.6	74.6	3.5	285.0	39.07	135.75

better dysglycemia risk classifiers, we constructed a log-logistic model as described above, incorporating also quadratic variables as predictors.

Performance metrics of the simple, quadratic and full logistic models using an optimal ROC-derived threshold probability are summarized in Table S12. Noteworthy, a comparison of the model performance indexes show that the impact of including many more explanatory variables is only marginally better in terms of performance metrics and this is done at the risk of increasing the risk of data overfitting. This observation justifies selecting the simplest model as the more statistically sound choice.

Table S12. Model attributes and performance metrics of diagnostic logistic models for classifying subjects according to their dysglycemic risk into healthy or non-healthy conditions, using the ROC classification threshold for classification

Performance metrics of diagnostic logistic models for assessing dysglycemia risk			
**Attribute or performance index**	**Simple model**	**Quadratic model**	**Full model**
Number of terms	7	10	41
Optimal ROC classification threshold	0.60	0.59	0.62
AUROC	0.84	0.85	0.92
Brier's score	0.16	0.15	0.11
Harrel's C index	0.84	0.85	0.92
Sensitivity	0.77	0.76	0.85
Specificity	0.74	0.77	0.83
False positive rate	0.26	0.23	0.17
False negative rate	0.23	0.24	0.15
Positive predictive value	0.85	0.87	0.91
Negative predictive value	0.62	0.63	0.74
Accuracy	0.76	0.77	0.85
Matthew's correlation coefficient (MCC)	0.49	0.52	0.67
Akaike Information Criterion (AIC)	1119	1116	877
F1 index	0.81	0.81	0.88
Pre-test probability (prevalence)	0.66	0.66	0.66
Diagnostic odds ratio [95% CI]	9.3 [7.1–12.3]	11.1 [8.4–14.8]	28.5 [20.6–39.5]
Positive likelihood ratio [95% CI]	2.9 [2.9–3.0]	3.4 [3.4–3.4]	5.0 [5.0–5.1]
Positive post-test probability	0.85	0.87	0.91
Negative likelihood ratio [95% CI]	0.32 [0.31–0.32]	0.30 [0.30–0.31]	0.18 [0.18–0.18]
Negative post-test probability	0.38	0.37	0.26
Prevalence threshold (ϕ_e)	0.18	0.19	0.21

4 MATHEMATICAL MODEL FOR THE G-I DYNAMICS

The synthetic model developed by Contreras et al. (2020) considers five compartments for describing glycemia-insulinemia (G-I) dynamics in an Oral Glucose Tolerance Test OGTT. Such compartments represent the amount (or concentration) of glucose in different compartments: in the stomach S, in the upper intestinal tract J (jejunum) and L (ileum), and in the bloodstream (glycemia) G. The last variable accounts for the insulinemia I. All magnitudes involved in the model are expressed in SI units; conversion factors for glycemia-related variables is 180 mg/dL = 10 mM, while for insulinemia 1 μU/mL = 6.945 pM. With the variables listed in Tab S13, the G-I dynamics are represented in Fig 1e in the main manuscript and Fig S7. The governing equations for the G-I dynamics according to the model of Contreras et al. (2020) are given by:

$$\frac{dS}{dt} = -k_{js}S, \qquad S(0) = D \tag{S1}$$

$$\frac{dJ}{dt} = k_{js}S - k_{gj}J - k_{jl}J, \qquad J(0) = 0 \tag{S2}$$

$$\frac{dL}{dt} = k_{jl}\varphi(t) - k_{gl}L(t), \qquad \varphi(t) = \begin{cases} 0, & \text{if } t < \tau \\ J(t-\tau), & \text{if } t \geq \tau \end{cases} \tag{S3}$$

$$\frac{dG}{dt} = -(k_{xg} + k_{xgi}I)G + G_{\text{prod}} + \eta\left(k_{gj}J + k_{gl}L\right), \qquad G(0) = G_b \tag{S4}$$

$$\frac{dI}{dt} = k_{xi}I_b\left(\frac{\beta^\gamma + 1}{\beta^\gamma\left(\frac{G_b}{\tilde{G}}\right)^\gamma + 1} - \frac{I}{I_b}\right) \tag{S5}$$

$$G_{\text{prod}} = \frac{k_\lambda}{\frac{k_\lambda}{G_{\text{prod}}(0)} + (G - G_b)}. \tag{S6}$$

Table S13. Model parameters

Parameter	Units	Physiological meaning
k_{js}	min^{-1}	Kinetic constant for stomach emptying
k_{gj}	min^{-1}	Kinetic constant for glucose absorption (jejunum)
k_{jl}	min^{-1}	Kinetic constant for glucose delivery from jejunum to ileum
τ	min	Time delay between glucose disappearance in jejunum and appearance in ileum
k_{gl}	min^{-1}	Kinetic constant for glucose absorption (jejunum)
k_{xg}	min^{-1}	Kinetic constant for basal glucose consumption
k_{xgi}	min^{-1} pM^{-1}	Kinetic constant for insulin-induced glucose consumption
η	(L^3)$^{-1}$	Bioavailability of the absorbed glucose
k_λ	mM^2 min^{-1}	Kinetic constant for hepatic glucose release rate
f_{gi}	min (L^3)$^{-1}$	Incretin action conversion factor
k_{xi}	min^{-1}	Kinetic constant for insulin degradation
β	-	Scale for insulin production saturation
γ	-	Scale for insulin production acceleration

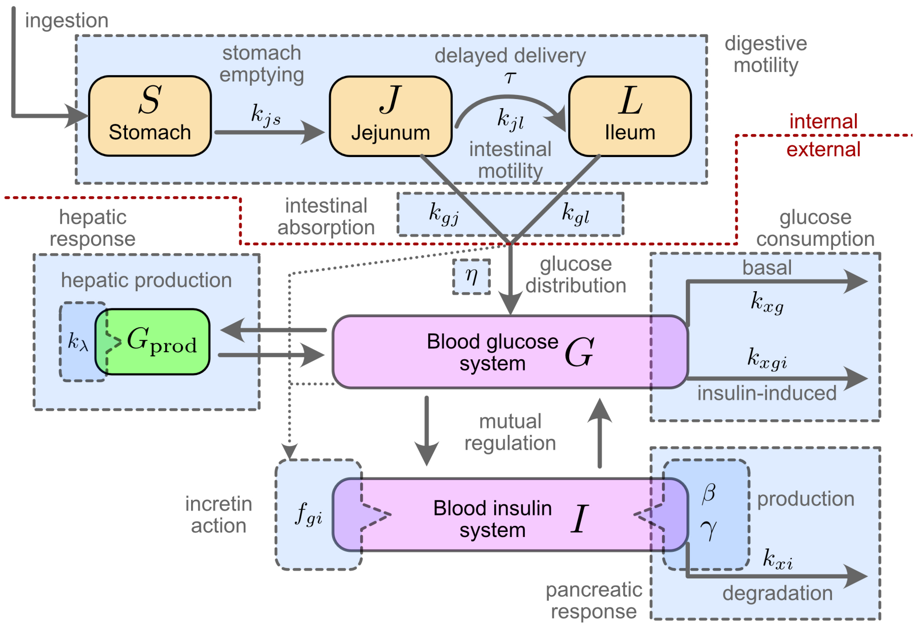

Figure S7. Glycemia-insulinemia (G-I) dynamics during an OGTT can be represented by a compact compartmental mathematical model (Contreras et al., 2020) capturing the main physiological processes behind glycemic control. Compartments are represented by colored boxes (orange for glucose pools in the lumen of the digestive system, magenta for blood concentrations and green for the liver compartment). Rate constants and parameters for each physiological process are indicated alongside their respective process arrows or compartments. General related processes, which account for subsystems, are shown as blue boxes. The red dotted line separates external parameters, which comprise physiological glucose processing up to glucose absorption into the body, from internal parameters, which includes all glucose processing inside the body after absorption.

4.1 Derivation of non-dimensional Π numbers (NDNs)

Non-dimensional analysis allows combining the contributions of a number of parameters involved in the studied dynamics (as the G-I dynamics), and to define combinations of such which control the overall behavior and stability of the system (Aston et al., 2011; Ghista, 2004; Dasi et al., 2008), independent of specific physical units. In our model (Contreras et al., 2020), there are 3 physical dimensions involved: time $[T]$, mass $[M]$, and length $[L]$. As the set of equations presented therein include 13 variables and 3 non-zero initial conditions, the number of dimensionless Π numbers that determine the dynamics of the system are 13, and they were constructed as follows. The natural scales that arise from the formulation of the problem are: D for mass, G_b for glucose and I_b for insulin concentrations. The chosen internal timescale was $t^* = \dfrac{f_{gj}}{\eta}$, which accounts for the ratio of incretin influence on pancreatic glucose perception vs. the specific rate of glucose appearance in the bloodstream due to intestinal absorption. Hence, we obtained the following non dimensional variables:

$$\sigma = \frac{S}{D} \qquad \chi = \frac{J}{D} \qquad \lambda = \frac{L}{D}$$

$$\mu = \frac{G}{G_b} \qquad \varepsilon = \frac{I}{I_b} \qquad T = \frac{t\eta}{f_{gj}} \qquad \rho = \chi + \Pi_G \lambda,$$

and the governing model equations now take the form:

$$
\begin{aligned}
\frac{d\sigma}{dT} &= -\Pi_S\sigma, && \sigma(0) = 1\\
\frac{d\chi}{dT} &= -\Pi_{JM}\chi + \Pi_S\sigma, && \chi(0) = 0\\
\frac{d\rho}{dT} &= \left(-\Pi_{JM} + \Pi_L\right)\chi\ \dots\\
&\cdots + \Pi_{MG}\chi(T-\Pi_\tau)H(T-\Pi_\tau) - \Pi_L\rho + \Pi_S\sigma && \rho(T) = \chi(T) \text{ if } T \le \tau_c\\
\frac{d\mu}{dT} &= -\left(\Pi_H + \Pi_X\left(\varepsilon - 1\right)\right)\mu + \frac{\Pi_H}{1 + \Pi_{HR}\left(\mu - 1\right)} + \Pi_{JD}\rho, && \mu(0) = 1\\
\frac{d\varepsilon}{dT} &= \Pi_I\left(\frac{\Pi_\omega + 1}{\Pi_\omega\left(\mu + \Pi_{JD}\rho\right)^{-\Pi_\gamma} + 1} - \varepsilon\right), && \varepsilon(0) = 1,
\end{aligned}
$$

Table S14. Model non-dimensional (NDN) Π numbers

Π number	Expression	Physiological process	Π number	Expression	Physiological process
Π_S	$\frac{k_{js}f_{gi}}{\eta}$	Stomach emptying	Π_I	$\frac{k_{xi}f_{gi}}{\eta}$	Basal pancreatic secretion
Π_M	$\frac{k_{lj}f_{gi}}{\eta}$	Intestinal motility	Π_W	β^γ	Pancreatic activity
Π_J	$\frac{k_{gj}f_{gi}}{\eta}$	Jejunal absorption	Π_γ	γ	Pancreatic acceleration
Π_G	$\frac{k_{gl}}{k_{gj}}$	Jejunal/ileal absorption ratio	β	β	Pancreatic saturation
Π_L	$\frac{k_{gl}f_{gi}}{\eta}$	Ileal absorption	Π_X	$\frac{k_{xgi}I_bf_{gi}}{\eta}$	Insulin activity load
Π_B	$\frac{k_{xgi}I_b}{k_{xg}}$	Relative insulin sensitivity	Π_R	$\frac{G_b^2\eta}{k_\lambda f_{gi}}$	Hepatic sensitivity
Π_C	$\frac{k_{xg}f_{gi}}{\eta}$	Basal glucose consumption	Π_D	$\frac{D\eta}{G_b}$	Distribution load
Π_N	$\frac{f_{gi}}{\tau\eta}$	Incretin activity load			

5 DISTRIBUTIONS OF PARAMETERS AND NDNS

Model parameters and NDNs obtained for subjects from the healthy and non-healthy cohorts show distinct highly-skewed leptokurtic distributions and ranges, and some among them present multimodal distributions. Except for f_{gi}, which was best described by a uniform distribution, shifted log-logistic functions best fitted all other model parameter and NDN distributions. This distribution family best approximated the data and simultaneously smoothed-out small local minor variations. The use of these fitted probability distributions allowed us to calculate confidence intervals for each parameter and NDNs for healthy and non-healthy subjects, reported in Tables S15 and S16. Since most distributions were very skewed, we show median instead of mean values and 90% two-tailed interval ranges for each parameter. Parameters and NDNs that have significantly different distributions between the healthy and non-healthy cohorts are highlighted in Tables S15 and S16. Blue represents parameters and NDNs that tend to decrease in the non-healthy group and red encodes parameters and NDNs that increase their value in non-healthy patients. Color intensity relates with the degree of significance of the differences, assessed by the Kolmogorov-Smirnov test p-value and the significance of the tail percentage differences. Interestingly, most differences are seen only at one distribution tail, while the other tail is invariant between healthy and non-healthy patients.

Table S15. Statistics of the model parameters for the healthy and non-healthy cohorts

MODEL PARAMETERS												
Physiological process	Parameter	Healthy median value	Range for healthy patients (90% confidence)	Non-healthy median value	Range for non-healthy patients (90% confidence)	Kolmogorov-Smirnov test p-value on samples and fitted distributions	Out-of-range tails in healthy patients (left/right tail)	Out-of-range tails in non-healthy patients (left/right tail)	p-value of tail differences (left/right tail) and direction of non-healthy change	Predictive threshold and direction of non-healthy change	Matthews correlation coefficient of the predictive threshold	Percentage of healthy/non-healthy patients out of threshold
Digestive motility	k_{js}	0.0372	0.0100<br>0.244	0.0282	0.00531<br>0.223	****	6.6%<br>6.4%	13.3% ****<br>5.1%	*** (↓)	0.0198 (↓)	0.141	21.8%<br>35.6%
	k_{lj}	0.0274	9.32×10^{-4}<br>0.464	0.0298	2.72×10^{-4}<br>0.626		6.6%<br>9.1% *	10.5% ****<br>13.3% ****	* (↓)	0.00399 (↓)	0.063	13.5%<br>18.5%
	τ	79.0	45.0<br>111	73.2	34.1<br>110	****	7.9%<br>7.1%	20.2% ****<br>7.3% *	**** (↓)	72.0 (↓)	0.165	33.8%<br>51.2%
Glucose absorption	k_{gj}	0.0430	0.0115<br>0.758	0.0522	0.00850<br>1.06		5.2%<br>8.6% *	8.0% *<br>14.4% ****	** (↑)	0.0680 (↑)	0.070	33.8%<br>41.1%
	k_{lg}	0.3412	0.00371<br>1.62	0.614	0.00253<br>1.67	*	2.2% ***<br>6.6%	3.4% *<br>11.5% ****	* (↑)	0.602 (↑)	0.332	26.9%<br>61.9%
Incretin potency	f_{gi}	2.97	0.124<br>5.33	2.90	0.0<br>5.41		5.9%<br>0% ****	7.5% *<br>0% ****		1.67 (↓)	0.014	23.2%<br>24.6%
Glucose challenge distribution	η	0.135	0.0145<br>6.97	0.279	0.0144<br>5.41	**	4.2%<br>1.0% ****	3.0% **<br>4.9%	**** (↑)	0.178 (↑)	0.384	31.6%<br>71.7%
	D	415	406<br>423	417	405<br>426	****	8.8% *<br>8.6% *	8.3% **<br>24.5% ****	**** (↑)	417 (↑)	0.073	36.6%<br>47.7%
Glucose uptake	k_{xg}	0.0534	1.26×10^{-5}<br>0.998	0.0788	6.44×10^{-5}<br>1.13	**	6.9%<br>7.6%	6.1%<br>11.1% ****		0.0581 (↑)	0.044	56.4%<br>61.1%
	k_{xgi}	5.26×10^{-5}	5.67×10^{-9}<br>0.00259	2.58×10^{-6}	5.68×10^{-12}<br>7.96×10^{-4}	****	7.9%<br>7.4%	19.5% ****<br>1.5% ****	**** (↓)<br>**** (↓)	9.41×10^{-6} (↓)	0.267	34.1%<br>62.3%
Hepatic response	k_{λ}	1.21	0.0207<br>69.7	1.66	0.00676<br>84.4		7.6%<br>10.6% **	7.7% *<br>15.0% ****		3.62 (↑)	0.049	30.5%<br>35.4%
Pancreatic response	k_{xi}	0.0560	0.0112<br>0.868	0.0586	0.0103<br>0.822		5.9%<br>12.0% ****	6.7%<br>10.5% ****		0.0631 (↑)	0.027	46.3%<br>49.3%
	γ	2.92	1.53<br>7.14	2.93	1.79<br>6.51	*	4.9%<br>6.6%	1.5% ****<br>7.2% *	* (↑)	2.12 (↑)	0.091	79.0%<br>85.9%
	β	6.27	1.51<br>121	5.39	1.57<br>113		5.9%<br>12.0% ****	4.4%<br>12.4% ****		2.33 (↑)	0.016	80.0%<br>81.4%
Effective control levels	G_b	4.74	4.10<br>5.29	5.03	4.27<br>6.13	****	4.2%<br>6.6%	1.5% ****<br>32.6% ****	* (↑)<br>**** (↑)	5.03 (↑)	0.302	18.6%<br>49.8%
	I_b	29.6	13.1<br>68.6	58.2	18.4<br>174	****	0% ****<br>5.9%	0% ****<br>40.5% ****	**** (↑)	47.9 (↑)	0.432	18.8%<br>64.2%

Table S16. Statistics of the model non-dimensional numbers for the healthy and non-healthy cohorts

MODEL NON-DIMENSIONAL NUMBERS												
**Physiological process**	**Non-dimensional number**	**Healthy median value**	**Range for healthy patients (90% confidence)**	**Non-healthy median value**	**Range for non-healthy patients (90% confidence)**	**Kolmogorov-Smirnov p-value on samples and fitted distributions**	**Out-of-range tails in healthy patients (left/right tail)**	**Out-of-range tails in non-healthy patients (left/right tail)**	**p-value of the tail differences (left/right tail) and direction of change**	**Predictive threshold and direction of non-healthy change**	**Matthews correlation coefficient of the predictive threshold**	**Percentage of healthy/non-healthy patients out of threshold**
**Digestive motility**	Π_S	0.357	0.00130<br>47.6	0.112	0.00105<br>16.0	****	2.0% ****<br>5.4%	5.9%<br>3.7%	** (↓) 	0.396 (↓)	0.175	47.2%<br>65.4%
	Π_M	0.389	2.12 × 10^{-4}<br>44.8	0.167	4.33 × 10^{-5}<br>37.0		4.4%<br>4.9%	8.3% **<br>4.8%	* (↓) 	0.156 (↓)	0.089	39.0%<br>48.4%
**Glucose absorption**	Π_G	3.98	0.0461<br>68.6	3.90	0.0427<br>158		4.7%<br>7.4%	6.1%<br>8.5% ***		13.5 (↑)	0.077	21.5%<br>28.8%
	Π_J	0.706	0.00175<br>56.6	0.349	6.85 × 10^{-4}<br>64.3		3.4%<br>8.4% *	5.8%<br>6.7%		0.175 (↓)	0.058	32.7%<br>38.5%
	Π_{JG}	1.85	0.0521<br>77.1	1.83	0.00709<br>58.5		6.1%<br>8.4% *	11.9% ****<br>5.6%	** (↓) 	0.131 (↓)	0.091	10.0%<br>16.8%
**Incretin potency**	Π_N	0.137	8.12 × 10^{-4}<br>3.84	0.0755	7.28 × 10^{-4}<br>1.86	*	2.0% ****<br>4.4%	5.4%<br>2.8% ***	** (↓) 	0.232 (↓)	0.142	54.6%<br>68.8%
**Glucose challenge distribution**	Π_D	12.1	1.20<br>550	22.7	1.25<br>445	***	3.4%<br>1.5% ****	2.8% ***<br>4.8%	 ** (↑)	15.4 (↑)	0.363	32.5%<br>70.3%
**Glucose uptake**	Π_B	0.0344	6.42 × 10^{-7}<br>159	8.75 × 10^{-4}	1.05 × 10^{-8}<br>40.1	****	5.2%<br>7.1%	14.8% ****<br>5.7%	**** (↓) 	0.00204 (↓)	0.206	33.8%<br>55.4%
	Π_X	0.0205	3.59 × 10^{-7}<br>0.395	4.41 × 10^{-4}	3.92 × 10^{-10}<br>0.138	****	6.4%<br>4.4%	15.4% ****<br>1.0% ****	**** (↓)<br>** (↓)	0.00866 (↓)	0.367	35.2%<br>73.1%
	Π_C	0.649	7.48 × 10^{-4}<br>3.74	0.640	0.00129<br>2.59		9.3% *<br>4.9%	10.6% ****<br>3.8%		2.26 (↓)	0.110	84.7%<br>91.9%
**Hepatic response**	Π_R	4.53	0.00933<br>134	5.86	0.0121<br>619	*	7.4%<br>6.6%	9.1% ***<br>14.4% ****	 **** (↑)	41.0 (↑)	0.105	14.2%<br>23.1%
**Pancreatic response**	Π_I	0.978	0.00871<br>19.0	0.467	0.00218<br>18.1	***	3.4%<br>4.4%	7.5% *<br>5.2%	** (↓) 	0.394 (↓)	0.108	34.5%<br>45.9%
	Π_ω	72.2	8.65<br>1.30 × 10^{7}	81.9	11.1<br>1.35 × 10^{7}		6.4%<br>10.1% **	1.9% ****<br>9.4% ***	** (↑) 	15.2 (↑)	0.073	82.7%<br>88.1%

Sensitivity analysis of fitted parameters

The parameter fitting procedure according to Contreras et al. (2020) was repeated at least by quintuplicate for each patient in order to check the fitting accuracy and uniqueness of parameter values. Furthermore, a sensitivity analysis was performed for each patient, in which the ten experimental measurements (five glycemia glycemia and five insulinemia points) were varied up and down by 10% of its original value, and parameters were fitted to these new points. This allowed us to determine parameters that were sensitive to errors in measurements and those that remained majorly unaffected. Conversely, this served to determine which parameters were well-defined by experimental points, and thus invariable, and which could be more variable for each patient.

This allowed us to find parameters for each patient that are unequivocally determined by all measured experimental points, *i.e.* they vary significantly when any small experimental point shift. In other words, these parameters closely depend on the form of the G-I curve profiles and, conversely, each patient's profile shapes are strongly determined by them. Therefore, we refer to these sensitive, unequivocally-determined parameters as ”profile-shaping parameters”. As expected, every patient showed a specific set of profile-shaping parameters responsible for determining their G-I curve shapes. Table 1, main text, summarizes individual contributions of each parameter to curve profile shaping in healthy and non-healthy cohorts. Radically different parameter contributions are observed when comparing influences within the same cohort. Moreover, significant shifts in profile-shaping parameters can be found when comparing the healthy and non-healthy groups. Notably, digestive motility and absorption-related parameters, namely k_{js}, k_{lj}, τ, k_{gj} and η, had a large effect on the observed dynamic profile. Additionally, jejunal absorption (k_{lj}) was more determinant for the curve shape than ileal absorption (k_{gj}).
Glucose distribution (η) was highly profile-determining while the perceived glucose challenge (D) was not determinant in approximately 25% of patients, which non-intuitively shows that for these patients curve profiles are independent from the external glucose challenge. In contrast, internal parameters (i.e. non-digestive parameters) were more sensitive to curve point location in a higher percentage of patients. More importantly, from a diagnostic point of view, curve shapes —and thus close relationships between glycemia and insulinemia explained by model dependencies— seem to be more determinant for the values of parameters of internal physiological processes. Glucose utilization parameters, especially insulin sensitivity (k_{xgi}), hepatic response (k_{λ}), and pancreatic response, mainly k_{xi}, are the most insensitive parameters, which might be a result of a collidor bias, since all patients considered were suspected to present impairments in the glucose-metabolism. Surprisingly, ileal absorption of glucose also seems to be highly dependent on the values of glycemia and insulinemia curves.
Specific glycemia and insulinemia profiles are determinant for the correct determination of model parameters. Consequently, physiological glycemic control can be closely associated with even small changes in glycemic and insulinemic curves during an OGTT. Notably, even though some relations between OGTT profiles and physiological aspects have been already described (Hulman et al., 2018), our model extends those results allowing us to link variations in OGTT curves to digestive and absorption parameters. When comparing parameter sensitivity between healthy and non-healthy cohorts (Table 1, main text), the percentage of patients with sensitive and insensitive parameters changes from healthy to non-healthy groups, although some parameters remain equally sensitive in both. The general trend is a decrease in parameter sensitivity for non-healthy patients, suggesting that parameters are more stringently determined in healthy patients. The above can be physiologically interpreted as a better load distribution among the different subsystems which contribute to the G-I control system, in healthy individuals. On the other hand, in non-healthy individuals control seem to over-rely on particular subsystems that become more

Table S17. Sensitivity analysis of the parameters expressed as the percentage of subjects for which a parameter was highly dependent (sensitive) from the exact value of G-I measurements

**Physiological process**	**Parameter**	**Percentage of subjects with sensitive parameter**	
		**Healthy cohort**	**Non-healthy cohort**
Digestive motility	k_{js}	83%	82%
	k_{lj}	71%	59%
	τ	87%	78%
Glucose absorption	k_{gj}	88%	79%
	k_{lg}	25%	15%
Incretin potency	f_{gi}	85%	73%
Glucose distribution	η	98%	97%
	D	76%	74%
Tisular glucose uptake	k_{xg}	68%	80%
	k_{xgi}	55%	32%
Hepatic response	k_{λ}	23%	19%
Pancreatic response	k_{xi}	54%	42%
	γ	100%	99%
	β	61%	58%
Effective control levels	G_b	98%	96%
	I_b	91%	79%

determinant to reach glycemic control in stricter ranges, while loosening the relevant ranges for non-determinant parameters. The higher sensitivity of k_{xg} and lower sensitivity of k_{xgi} in non-healthy patients might indicate a higher relative reliance on insulin-independent glucose consumption for glycemic control in those patients. Remarkably, this implies that individuals with glucose control impairment are likely to be less responsive to G-I interactions.

Table S18. Polyphenolic composition of Delphinol used in this study

**Other polyphenols**	**% w/w**	**Anthocyanin composition**	**% w/w**
Gallic acid	1.45	Delphinidin-3-sambubioside-5-glucoside	7.52
Catechin	0.21	Delphinidin-3,5-di-glucoside	11.98
Epicatechin	2.90	Cyanidin-3-sambubioside-5-glucoside	2.93
Vanillic acid	0.43	Cyanidin-3,5-di-glucoside	2.41
Protocatechuic acid	0.28	Delphinidin-3-sambubioside	3.01
Kaempferol	0.03	Delphinidin-3-glucoside	9.02
Myricetin	0.24	Cyanidin-3-sambubioside	1.06
Rutin (quercetin-3-rutinoside)	0.76	Cyanidin-3-glucoside	1.49
Quercetin	0.51	**Total delphinidins**	**31.53**
Ellagic acid	11.79	**Total anthocyanins**	**39.42**
Catechin derivatives (catechin equivalents)	17.79		
Flavonol derivatives (kaempferol equivalents)	0.40		
Flavanone derivatives (hesperetin equivalents)	0.43		
Other unidentified peaks (catechin equivalents)	1.13		
**Total other polyphenols**	**38.33**		

REFERENCES

Contreras S, Medina-Ortiz D, Conca C, Olivera-Nappa Á. A novel synthetic model of the glucose-insulin system for patient-wise inference of physiological parameters from small-size ogtt data. *Frontiers in bioengineering and biotechnology* **8** (2020) 195.

Aston PJ, Derks G, Raji A, Agoram BM, van der Graaf PH. Mathematical analysis of the pharmacokinetic–pharmacodynamic (pkpd) behaviour of monoclonal antibodies: predicting in vivo potency. *Journal of theoretical biology* **281** (2011) 113–121.

Ghista DN. Physiological systems'numbers in medical diagnosis and hospital cost-effective operation. *Journal of Mechanics in Medicine and Biology* **4** (2004) 401–418.

Dasi LP, Pekkan K, Katajima HD, Yoganathan AP. Functional analysis of fontan energy dissipation. *Journal of biomechanics* **41** (2008) 2246–2252.

Hulman A, Witte DR, Vistisen D, Balkau B, Dekker JM, Herder C, et al. Pathophysiological characteristics underlying different glucose response curves: a latent class trajectory analysis from the prospective egir-risc study. *Diabetes Care* **41** (2018) 1740–1748.
